# Moving toward wellbeing: physical activity and quality of life in individuals with physical disabilities in Saudi Arabia

**DOI:** 10.3389/fpsyg.2025.1684083

**Published:** 2025-11-03

**Authors:** Majed M. Alhumaid, Mohamed A. Said

**Affiliations:** ^1^Department of Physical Education, College of Education, King Faisal University, Al-Ahsa, Saudi Arabia; ^2^King Salman Center for Disability Research, Riyadh, Saudi Arabia

**Keywords:** quality of life, physical disabilities, physical activity, structural equation modeling, self-perceived health, socioeconomic factors

## Abstract

**Background and objectives:**

This study examined the associations between quality of life (QoL) and physical activity (PA), self-perceived health and fitness, and sociodemographic characteristics among individuals with physical disabilities (IWPDs) in Saudi Arabia.

**Materials and methods:**

A total of 230 IWPDs aged 18 years and older participated in the study, comprising 133 males (57.8%) and 97 females (42.2%). QoL was assessed using the World Health Organization Quality of Life–Disabilities module, while PA levels were measured using the Arabic version of the Physical Activity Scale for Individuals with Physical Disabilities. Structural equation modeling was used to examine the relationships of overall PA and its specific domains—household, recreational, vocational, and home-repair activities—with overall QoL and its subdomains.

**Results:**

Self-perceived health and fitness were identified as the strongest correlates of both overall and domain-specific QoL. Education and income were positively associated with multiple QoL components—participants with higher education levels reported significantly greater Discrimination (β = 0.141), Autonomy (β = 0.236), and Inclusion/Participation (β = 0.29) but lower social relationships (β = −0.336). While total PA was not significantly associated with overall QoL, specific PA domains showed nuanced effects; For example, household activities were positively associated with the psychological domain (β = 0.25), social relationships (β = 0.542), environmental domain (β = 0.149), and autonomy-related domain (β = 0.531), but were negatively associated with physical health (β = −0.336) and inclusion/participation (β = −0.399). In contrast, home repair activities exhibited the opposite pattern. Sex differences were also observed, with men reporting lower QoL than women across several domains.

**Conclusions:**

These findings underscore the relevance of health, education, employment, and psychosocial factors for QoL among IWPDs and provide insights that may inform future research as well as evidence-based health and disability policy planning in Saudi Arabia.

## 1 Introduction

Quality of life (QoL) is a multifaceted concept that includes an individual's physical health, psychological wellbeing, social relationships, and environmental interactions (Schalock et al., 2002). The World Health Organization (WHO) defines QoL as “an individual's perception of their position in life in the context of the culture and value systems in which they live, and in relation to their goals, expectations, standards and concerns” (WHOQOL Group, 1995). This multidimensional perspective considers QoL as a crucial indicator of overall health, particularly for those with chronic health conditions or disabilities. The broad and individual nature of QoL makes it a valuable metric for assessing individual adaptation to and management of physical impairments.

Physical disabilities, which may arise due to congenital conditions, injuries, or diseases, are associated with significant restrictions in mobility and independence. These impairments may be linked to various aspects of life, including self-esteem, body image, and engagement in family and community activities (Krause, 2003; Cegarra et al., 2023; Livneh and Antonak, 2005). Research has consistently indicated that individuals with physical disabilities (IWPDs) exhibit lower QoL, especially in the Physical Health and Environmental domains (Post and van Leeuwen, 2012; Sulaimani et al., 2023; Kim et al., 2025). Therefore, we hypothesize that IWPDs will report lower QoL, particularly in areas directly associated with functional limitations (H1).

In addition to physical impairments, individuals with disabilities frequently encounter psychological and social barriers, including stigma, exclusion, and limited access to education and employment opportunities. These challenges may be associated with an increased likelihood of mental health conditions, such as depression and anxiety, which may in turn be linked to lower QoL (Skevington et al., 2004; Emerson, 2003; Jung et al., 2021). Some studies indicate that specific domains, such as spiritual or family life, may remain relatively intact or even function as protective factors among individuals with disabilities (Albrecht and Devlieger, 1999; Gignac et al., 2002).

Physical activity (PA) is increasingly recognized as a significant factor associated with quality of life, especially in individuals with disabilities. Regular PA is positively associated with better physical health, lower pain, improve mood, and increase perceived competence and autonomy (Kroll et al., 2006; Tomasone et al., 2013; Rimmer and Rowland, 2008). Research indicates that PA can mitigate negative stereotypes by enhancing independence and self-efficacy, as well as promoting social inclusion and peer support (Jaarsma et al., 2014; Kiuppis, 2016; Blauwet and Willick, 2012). Therefore, we hypothesize that higher levels of PA will be positively associated with higher QoL across various domains (H2).

Participation in PA is notably low among IWPDs, primarily due to environmental, social, and psychological barriers. IWPDs often encounter barriers to inclusive exercise programs, adapted facilities, and motivational support. In numerous countries, these challenges are exacerbated by insufficient policy support and inadequate infrastructure (Shields et al., 2012; Martin Ginis et al., 2016). Therefore, we hypothesize that daily PA levels among IWPDs in Saudi Arabia will fall within the low-to-moderate range (H3).

### 1.1 Study context: Saudi Arabia

In Saudi Arabia, individuals with physical disabilities represent a significant yet underrepresented group in public health and rehabilitation research. The latest disability survey conducted by the General Authority for Statistics in 2017 (General Authority for Statistics, 2017) indicates that around 7.1% of Saudis aged 15 years or older experience some form of disability, with physical and mobility impairments being the most frequently reported. In addition, more than 60% of these individuals encounter challenges in executing daily living activities, including walking, standing, or self-care, underscoring the significant functional limitations present in this population.

Saudi Arabia has made significant policy advancements, including the ratification of the United Nations' Convention on the Rights of Persons with Disabilities, the enactment of the Saudi Disability Law, and the incorporation of disability rights in Vision 2030. However, notable gaps remain, including restricted accessibility in the built environment, inadequate transportation infrastructure, and a lack of inclusive PA and sports programs, especially for women and individuals in rural areas (Alquraini, 2020; Saleh and Alturif, 2025).

There is a lack of empirical studies examining the relationship between PA and QoL among individuals with disabilities in Saudi Arabia. Recent research has begun to shed light on this issue. For instance, Alhumaid et al. (2024c) identified significant associations between PA, self-perceived fitness (SP-fitness), and sociodemographic factors with life satisfaction among IWPDs. In addition, a qualitative study by Alhumaid et al. (2024a) revealed that participation in para-sports was associated with higher social inclusion and sense of identity. Moreover, Said and Alhumaid (2024) found that PA levels varied significantly among individuals with disabilities according to disability type, use of mobility aids, and demographic characteristics. Nonetheless, despite significant efforts, there is a lack of nationally representative quantitative studies employing validated instruments to assess both PA and QoL.

The significance of this gap is underscored by Saudi Arabia's geographic and cultural diversity, which may affect the perceptions and engagement of IWPDs in PA. Comprehensive data are essential to designing effective and inclusive health promotion strategies that cater to the physical, emotional, and social needs of this population.

### 1.2 Study aims

This study assessed PA levels and QoL among IWPDs living in various regions of Saudi Arabia. It used two validated instruments: (1) the Arabic version of the Physical Activity Scale for Individuals with Physical Disabilities (PASIPD-AR) to assess PA levels, and (2) the Arabic version of the WHO Quality of Life–Disabilities module (WHOQOL-DIS-AR) to assess QoL. In addition to investigating the association between PA level and QoL (overall and components), this study aimed to identify demographic, health-related, and activity-specific factors associated with QoL among IWPDs. Therefore, this study adds to the global discussion on health equity and disability, particularly in the Middle East, by offering nationally relevant data.

## 2 Materials and methods

### 2.1 Data collection and participants

This study used an online survey to recruit individuals with physical disabilities between March 1 and April 30, 2025. A total of 250 individuals with physical disabilities were invited to participate via phone, email, and WhatsApp from disability centers and organizations in Saudi Arabia's six administrative regions: Western, Northern Border, Jazan, Tabuk, Riyadh, and Hail.

The inclusion criteria were as follows: age 18 years or older, literate in Arabic, have a medically documented physical disability certified by an official certificate from the Saudi Ministry of Human Resources and Social Welfare, and agree to participate in our study. The exclusion criteria were as follows: having a serious cognitive or psychological disorder that could affect response accuracy, and incomplete or inconsistent responses.

All participants received an invitation message outlining the study's purpose, procedures, inclusion criteria, and participation incentive. A hyperlink directed them to the Google Forms survey platform. On the landing page, participants were required to review and digitally agree to an informed consent form before accessing the questionnaires. They were informed that their participation was voluntary, the survey would take around 25 min to complete, and they could withdraw at any time without repercussion. Upon submission, the data was downloaded and checked for accuracy and completeness. No personal identification information was collected.

Of the 250 IWPDs who accessed the survey, 243 submitted complete questionnaires. Following data screening, 13 questionnaires were excluded: ten due to missing responses for more than 20% of the total survey items (i.e., >14 out of 69 items) (Skevington et al., 2004); one due to an invariant response pattern (identical answers across all items); and two due to implausible demographic inconsistencies (e.g., mismatch between reported age and marital status). Examination of missing data patterns indicated that item-level missingness among retained cases was minimal (< 2%) and randomly distributed. Therefore, listwise deletion of the 13 invalid cases was deemed appropriate. The final analytic sample included 230 valid responses (133 males, 57.8%; 97 females, 42.2%). The average age of participants was 38.4 years (±10.2), with males at 39.1 (±9.8) and females at 37.5 (±10.7) years. This resulted in a final response rate of 92.0%.

The study protocol was approved by the Research Ethics Committee of King Faisal University (approval no. KFU-REC-2024-OCT-ETHICS2863), and all procedures were conducted in accordance with the ethical standards outlined in the Declaration of Helsinki (World Medical Association, 2013). Data confidentiality and security were maintained throughout the study.

### 2.2 Instruments

Data was collected using three structured questionnaires, which included a total of 69 items. The first instrument consisted of 13 items to collect demographic and health-related data, including height, weight, self-perceived health (SP-health), SP-fitness, and fundamental sociodemographic characteristics. The second instrument was the PASIPD-AR (Alhumaid et al., 2024b), which comprised 13 items designed to evaluate participants' physical activity levels and the types of activities they performed. The third instrument was the Arabic version of the WHOQOL-DIS-AR (Said and Alhumaid, in press), which comprised 38 items, including 25 from the brief version of the 100-item WHOQOL questionnaire (WHOQOL-BREF) designed to evaluate various dimensions of QoL, and 13 disability-specific items, referred to as the Disabilities module, designed to assess Quality of life aspects unique to disabilities not addressed by the basic scale.

#### 2.2.1 Demographic questionnaire

This instrument collected data on participants' age, sex, education, particular physical limitations, types of physical aid utilized, height, weight, marital status, occupation, average family income, and their SP-health and SP-fitness levels. The latter two variables were evaluated on a three-point Likert scale (SP-health: bad, medium, or good; SP-fitness: low, moderate, or high) based on participants' responses to two questions concerning their health and fitness.

#### 2.2.2 PASIPD-AR

The Physical Activity Scale for Individuals with Physical Disabilities is a modified and verified Arabic adaptation of the original PASIPD created by Washburn et al. (2002). This version was specifically tailored for application in Saudi Arabia, with its psychometric qualities assessed by Alhumaid et al. (2024b). The assessment was conducted in alignment with the Consensus on Exercise Reporting Template (CERT) (Slade et al., 2016) to improve the clarity and replicability of the physical activity data.

Confirmatory factor analysis (CFA) conducted in a cohort of 206 IWPDs substantiated the scale's construct validity, with factor loadings ranging from 0.583 to 0.889, collectively accounting for 61.62% of the total variance. Its internal consistency was deemed adequate, with a Cronbach's alpha of 0.694 for the entire scale and 0.420–0.619 for the subscales, thereby supporting its application in population-level evaluations of PA within this context.

The PASIPD-AR comprises 13 items that evaluate PA across four domains—Leisure, Home, Occupation/Transportation, and Inactivity—based on activities conducted in the preceding seven days. Each item documents the frequency (never/seldom = 1–2 days/week, occasionally = 3–4 days/week, often = 5–7 days/week) and duration (< 1 h, 1 to < 2 h, 2–4 h, or >4 h per day) of activity participation.

Unlike the original English version of the PASIPD, the PASIPD-AR incorporates four latent factors as opposed to the original five: Factor 1 includes home repairs, lawn mowing, and garden work activities (HRA; items 9, 10, 11); Factor 2 involves household activities (HHA; items 7, 8, 12); Factor 3 encompasses light to vigorous sports and recreational activities (SRA; items 3, 4, 5, 6); and Factor 4 encompasses occupational and transportation activities (OTA; items 2 and 13). The first item (item 1) functions as a contextual reference but is excluded from the scoring process. The total PASIPD-AR scores are calculated by multiplying the stated average daily hours for each activity by its corresponding metabolic equivalent of task (MET) value. They vary from 0.0 to 199.5 MET-h/day, with larger scores indicating increased levels of PA across several life domains.

#### 2.2.3 WHOQOL-DIS-AR

The Arabic version of the WHO Quality of Life–Disabilities module is a culturally adapted and translated version of the original English version of the WHOQOL-DIS questionnaire designed for individuals with disabilities. It was specifically adapted to the Saudi social context, and its psychometric qualities have been assessed by Said and Alhumaid (2025). In accordance with guidelines for transparent reporting of patient-reported outcomes (PROs) (Calvert et al., 2013), we present a comprehensive description of the PRO instrument (WHOQOL-DIS-AR), encompassing its cultural adaptation, psychometric properties, and the management of missing data, to enhance interpretation and critical evaluation. The instrument consists of 38 items, reduced from the original 39-item scale due to the exclusion of Item 21, which pertains to sexual satisfaction, based on considerations of cultural sensitivity.

The WHOQOL-DIS-AR consists of two interconnected components: the WHOQOL-BREF and the Disabilities module. Both exhibited robust construct validity, as verified by retroactive principal component analysis and CFA, performed on two independent samples of 188 IWPDs. While the WHOQOL-BREF maintained a four-domain framework, the Disabilities module was most accurately depicted by a three-facet model. All items satisfied the standard factor loading criterion of ≥0.40, with the total variance explained reaching 65.36% for the WHOQOL-BREF and 73.08% for the Disabilities module.

The internal consistency was elevated across all domains of the WHOQOL-DIS-AR. The WHOQOL-BREF had a Cronbach's alpha of 0.901 for the entire scale and 0.845–0.909 for the subscales, indicating exceptional reliability. The Disabilities module demonstrated satisfactory reliability, with a Cronbach's alpha of 0.761 for the entire scale and 0.643–0.948 for the subscales, indicating acceptable to outstanding internal consistency. These findings support the appropriateness of the WHOQOL-DIS-AR for evaluating QoL in IWPDs in Saudi Arabia.

The WHOQOL-BREF assesses four primary domains: (1) Physical Health (seven items), encompassing energy, mobility, pain, sleep, daily activities, medication dependency, and work capacity; (2) Psychological (six items), encompassing self-esteem, body image, positive and negative emotions, focus, and personal beliefs; (3) Social Relationships (two items), encompassing personal relationships and social support; and (4) Environment (eight items), encompassing safety, financial resources, access to healthcare and information, recreation, residential conditions, and transportation. It is important to note that the cultural adaptation procedure for the Arabic context necessitated a rigorous assessment of item relevance. Notably, in addition to the removal of Item 21 from the original instrument, Item 14, which is part of the Environmental domain and deals with the degree of satisfaction with opportunities for leisure activity, had low factor loading and explained variance, raising concerns about its conceptual clarity and cultural relevance in this setting. The current investigation of the WHOQOL-DIS-AR thus gives an opportunity to investigate the stability of these measurement issues in a particular disability group.

The Disabilities module comprises 13 additional items intended to document disability-specific experiences. The first item (item 26) is a comprehensive item evaluating the total perceived influence of disability on daily living; it was excluded from the analysis. The remaining 12 items (27–38) were categorized into three facets: (1) Discrimination (items 27–29), which assesses experiences of disrespect, adverse attitudes, and inequitable treatment; (2) Autonomy (items 30–32), which assesses constraints in decision-making and self-sufficient operation; and (3) Inclusion/Participation (items 33–38), which evaluates societal integration, encompassing participation in education, employment, community engagement, and access to services.

All items were evaluated using a 5-point Likert scale (1 = very dissatisfied/very poor/not at all to 5 = very satisfied/very good/extremely), and negatively worded items were reverse-scored. A standardized transformation formula was used to convert mean raw scores to a 0–100 scale for each QoL subcomponent, with higher scores indicating better perceived QoL. This approach improves interpretability and facilitates the identification of domains most affected by disability-related challenges.

### 2.3 Statistical analysis

Statistical analyses were performed using the R statistical software (version 4.3.1), utilizing the *lavaan* package (version 0.6–19) for CFA and structural equation modeling (SEM), along with other packages including psych, car, and performance for data diagnostics. Descriptive statistics, including means ± standard deviations and medians (interquartile ranges), were calculated for all study variables to summarize the characteristics of the study sample. Before estimating the model, the assumptions of multivariate analysis were assessed. Outliers were identified using the Mahalanobis distance and addressed through winsorization to minimize their impact while maintaining the integrity of the data structure. Bivariate correlations were computed to examine relationships between key study variables. The full correlation matrix is provided in Supplementary material 2. Variance inflation factors were used to evaluate multicollinearity, revealing that all correlates remained below the conservative threshold of 5. Normality was evaluated through the analysis of skewness and kurtosis, complemented by visual examination of Q–Q plots. Given the ordinal nature of the WHOQOL-DIS-AR items and observed non-normality in some variables, we employed robust estimation methods to ensure valid parameter estimates and accurate standard errors. Residual plots were examined and revealed no significant heteroscedasticity.

A Confirmatory Factor Analysis (CFA) was conducted to validate the established seven-factor structure of the Arabic version of the WHO Quality of Life–Disabilities module within our sample. Model fit was assessed using a suite of standard indices: the Comparative Fit Index (CFI), Tucker-Lewis Index (TLI), and Normed Fit Index (NFI), with values ≥0.90 indicating acceptable fit, and ≥0.95 indicating excellent fit. The Root Mean Square Error of Approximation (RMSEA) was interpreted with values < 0.08 indicating good fit and values up to 0.12 deemed acceptable in more complex models (Xia and Yang, 2019). The Standardized Root Mean Square Residual (SRMR) was used with a cutoff of < 0.08 for good fit. Domains were retained solely when model fit and factor loadings indicated construct validity.

Following CFA validation, a series of SEMs were estimated to investigate associations between predictors and QoL outcomes. An initial model tested overall QoL as a latent construct, and subsequent models examined each of the seven QoL components separately: Physical Health, Psychological, Social Relationships, and Environment domains, as well as Discrimination, Autonomy, and Inclusion/Participation facets. The predictor set was consistent across all models and included age, sex, BMI, education, occupation, type of disability, social relationships, income, use of disability aids, SP-health, SP-fitness, and physical activity (operationalized either as total PASIPD-AR or its four subscales). Because the indicator variables were ordinal, all models were estimated using the Diagonally Weighted Least Squares (DWLS) estimator, which provides robust estimates under non-normal conditions.

To address the issue of multiple comparisons, the analysis explicitly considered the increased familywise Type I error rate. However, no universal correction (e.g., Bonferroni) was applied, as such procedures would have been overly conservative and could obscure theoretically meaningful associations by inflating Type II errors. Instead, analyses were guided by predefined hypotheses focused on distinct QoL constructs, rather than exploratory testing. Furthermore, the emphasis was purposely switched from a binary focus on statistical significance to an assessment of effect size magnitude and estimate precision. Standardized regression coefficients (β) are therefore presented as effect sizes throughout the manuscript, and 90% confidence intervals are reported in Supplementary files to facilitate assessment of parameter precision. In accordance with contemporary standards for clear reporting, p-values are displayed to three decimal places and analyzed in conjunction with effect sizes and confidence intervals. Effect sizes were categorized as Negligible (|β| < 0.10), Small (0.10 ≤ |β| < 0.20), Medium (0.20 ≤ |β| < 0.50), and Large (|β| ≥ 0.50). Statistical evidence was assessed at α = 0.05, highlighting the strength, direction, and precision of associations instead of strict significance thresholds.

## 3 Results

### 3.1 Participants' characteristics

The study sample consisted of 230 individuals with physical disabilities (133 males, 97 females) with a mean age of 36.90 ± 15.38 years (Table 1). Demographically, a large majority of participants reported being either single (42.6%) or married (41.7%). Education levels varied, with secondary school (34.8%) and university degrees (21.7%) being the most common attainments. Socioeconomically, most participants were not formally employed (80.4%) and had a monthly income below 5,000 SAR (71.3%). Regarding disability characteristics, the most common types were “other physical conditions” (40.9%), polio (18.7%), and paraplegia (18.3%). Most participants used assistive devices, primarily wheelchairs (42.2%), while 38.7% used no mobility aids. Participants' self-perceptions of their health and fitness were generally low. A majority rated their health as “medium” (67.0%) and their fitness level as “low” (59.6%); only 13.9% and 5.7% rated their health and fitness as “good” or “high,” respectively.

**Table 1:** Participants' categorical characteristics (*N* = 230).

**Characteristic**	**Category**	***n***	**%**
Sex	Male	133	57.8%
	Female	97	42.2%
Marital status	Single	98	42.6%
	Married	96	41.7%
	Divorced	14	6.1%
	Widow	14	6.1%
	Refused to declare	8	3.5%
Highest education level	Primary	64	27.8%
	Middle	36	15.7%
	Secondary	80	34.8%
	University	50	21.7%
Occupation	No occupation	185	80.4%
	Public sector	21	9.1%
	Other	24	10.4%
Monthly income (SAR)	< 5,000	164	71.3%
	5,000–10,000	33	14.3%
	10,001–15,000	10	4.3%
	0 (no income)	23	10.0%
Type of disability	Polio	43	18.7%
	Paraplegia	42	18.3%
	Leg/foot amputation	18	7.8%
	Cerebral palsy	22	9.6%
	Muscular dystrophy	11	4.8%
	Others	94	40.9%
Disability aid	None	89	38.7%
	Wheelchair	97	42.2%
	Crutches	25	10.9%
	Cane	19	8.3%
SP-health	Bad	44	19.1%
	Medium	154	67.0%
	Good	32	13.9%
SP-fitness	Not active	80	34.8%
	Moderately active	137	59.6%
	Active	13	5.7%

SAR, Saudi Arabian Riyal; SP, self-perceived.

Regarding the participants' physical characteristics (Table 2), the mean weight was 65.83 ± 22.03 kg, the mean height was 153.95 ± 26.64 cm, and the mean BMI was 27.76 ± 8.67 kg/m^2^. Regarding physical activity, the PASIPD-AR factors—HRA, HHA, SRA, and OTA—exhibited low mean scores, with a total PASIPD-AR score of 15.03 ± 25.82 MET-hours/day that support H3. Regarding QoL, scores varied significantly across the seven WHOQOL-DIS-AR components, with the highest mean score observed for the Social Relationships domain (66.00 ± 26.76), followed by the Psychological domain (56.25 ± 21.58), and the lowest mean score observed for the Environmental domain (46.00 ± 20.97), indicating possible difficulties in this area. The mean total WHOQOL-DIS-AR score was 57.75 ± 24.24, indicating a moderate level of perceived QoL among Saudi IWPDs. Using the equal-interval classification method, participants' QoL ratings were distributed as follows: very good (18.7%), good (26.5%), moderate (29.6%), poor (15.7%), and very poor (9.6%). Overall, most participants (54.8%) reported QoL levels ranging from moderate to good, indicating a generally positive self-perception of QoL and partially refuting H1.

**Table 2:** Participants' continuous characteristics (*N* = 230).

**Variable**	**Mean ±SD**	**Min**	**Max**
Age (years)	36.90 ±15.38	17.00	73.00
Weight (kg)	65.83 ± 22.03	21.00	142.00
Height (cm)	153.95 ± 26.64	42.00	181.00
BMI (kg/m^2^)	27.76 ± 8.67	17.58	55.00
PASIPD-AR: HRA (MET-hours/day)	2.78 ± 7.16	0.00	51.48
PASIPD-AR: HHA (MET-hours/day)	2.57 ± 5.03	0.00	30.03
PASIPD-AR: SRA (MET-hours/day)	5.84 ± 11.15	0.00	75.07
PASIPD-AR: OTA (MET-hours/day)	3.84 ± 10.25	0.00	75.12
Total Physical Activity (MET-hours/day)	15.03 ± 25.82	0.00	169.23
WHOQOL-DIS-AR: Physical Health	49.5 ± 21.16	3.5	85.75
WHOQOL-DIS-AR: Psychological	56.25 ± 21.58	8.25	100.00
WHOQOL-DIS-AR: Social Relationships	66.00 ± 26.76	0.00	100.00
WHOQOL-DIS-AR: Environment	46.00 ± 20.97	0.00	93.75
WHOQOL-DIS-AR: Discrimination	53.00 ± 29.17	0.00	100.00
WHOQOL-DIS-AR: Autonomy	50.00 ± 16.26	0.00	100.00
WHOQOL-DIS-AR: Inclusion/Participation	49.00 ± 18.67	16.75	83.25
Overall QoL	57.75 ± 24.24	8.25	100.00

BMI, body mass index; HHA, household activities; HRA, home repair activities; Max, maximum; MET, metabolic equivalent; Min, minimum; OTA, occupational and transportation activities; PA, physical activity; PASIPD-AR, Arabic version of the Physical Activity Scale for Individuals with Physical Disabilities; QoL, quality of life; SD, standard deviation; SRA, sports and recreational activities; WHOQOL-DIS-AR, Arabic version of the World Health Organization Quality of Life–Disability module.

### 3.2 CFA of the WHOQOL-DIS-AR and preliminary bivariate analysis

Before conducting Structural Equation Modeling, a Confirmatory Factor Analysis was conducted to evaluate the fit of the previously validated seven-factor structure of the WHOQOL-DIS-AR among IWPDs in Saudi Arabia (Table 3). The model demonstrated an acceptable overall fit [χ^2^_(539)_ = 1976.43, *p* < 0.001], significantly outperforming the baseline model [χ^2^_(595)_ = 34,715.83, *p* < 0.001]. The fit indices indicated adequate model performance: CFI = 0.958, TLI = 0.953, normed fit index (NFI) = 0.943, and RMSEA = 0.108 (90% confidence interval [CI]: 0.103–0.113), which falls within the acceptable threshold of 0.12. Although the SRMR of 0.117 slightly exceeded conventional cutoffs, all factor loadings were statistically significant (*p* < 0.001), with standardized coefficients ranging from 0.533 to 2.335, indicating strong item–factor relationships.

**Table 3:** Summary of model fit indices for the WHOQOL-DIS-AR.

**Fit index**	**Value**	**Threshold**	**Interpretation**
χ^2^ (d*f* = 539)	1,976.43	–	Significant, large sample
CFI	0.958	≥0.95	Excellent fit
TLI	0.953	≥0.95	Excellent fit
NFI	0.943	≥0.90	Good fit
RMSEA (90% CI)	0.108 (0.103–0.113)	≤ 0.08	Marginal fit
SRMR	0.117	≤ 0.08	Slightly above threshold
AVE (Factors 1–7)	0.446–0.944	≥0.50	Mostly adequate fit
*R* ^2^	0.122–0.980	–	High item reliability
Factor correlations	–	–	Mostly positive, some negative

AVE, average variance extracted; CFI, comparative fit index; df, degrees of freedom; NFI, normed fit index; TLI, Tucker–Lewis index; R^2^, coefficient of determination; RMSEA, root mean square error of approximation; SRMR, standardized root mean square residual.

The average variance extracted (AVE) for most factors exceeded 0.50 (range: 0.446–0.944), demonstrating acceptable convergent validity in this sample. The coefficient of determination (*R*^2^) for item-level variances was generally high (e.g., Item 19: *R*^2^ = 0.951, Item 36: *R*^2^ = 0.980), supporting item reliability. The model also revealed mostly positive and significant inter-factor correlations, although several negative covariances (e.g., between factors 1 and 5) emerged and merit further theoretical consideration. These results confirm the structural adequacy of the WHOQOL-DIS-AR among IWPDs in Saudi Arabia, providing a validated measurement model for examining associations between PA and QoL.

Nevertheless, *post-hoc* evaluation highlighted areas of localized misfit that align with the instrument's adaptation history. In the Physical Health domain, Item 3 (physical discomfort) showed a notably low R^2^ value, while in the Environmental domain, the AVE fell below the recommended threshold. These findings are parallel to earlier validation studies. In particular, Item 14, which asks about opportunities for leisure activities, previously showed weak performance in the Arabic WHOQOL-BREF and again emerged as a problematic indicator contributing to misfit in the Environmental domain. This recurring issue points to cultural challenges in capturing the construct of leisure within the Saudi context. Similarly, the earlier exclusion of Item 21 (sexual satisfaction) for cultural sensitivity reasons illustrates that contextual modifications are sometimes necessary when applying the WHOQOL in Arabic-speaking populations. Thus, the observed discrepancies are not anomalies but reflect systemic difficulties in achieving full metric equivalence, particularly for domains where cultural norms and environmental barriers may influence item interpretation. More comprehensive model fit indices and supplementary construct validity measures are provided in Supplementary material 1.

#### 3.2.1 Preliminary bivariate analysis

Bivariate correlations were evaluated to help inform the subsequent SEM. This analysis found a favorable correlation between household activities and physical health (*r* = 0.39, *p* < 0.001). However, the PA domains were substantially intercorrelated (e.g., household activities and home maintenance, *r* = 0.80; see Supplementary material 2 for the full matrix). This pattern of multicollinearity is a known requirement for suppression effects in multivariate models, and it is crucial for understanding the structural results that follow.

### 3.3 SEM: Associations with overall QoL among IWPDs

To examine the factors associated with overall quality of life among individuals with physical disabilities, two SEMs were tested using DWLS estimation. In both models, overall QoL was specified as a latent construct measured by the three general items of the WHOQOL-DIS-AR: item 1, satisfaction with physical health; item 2, overall QoL perception; and item 26, satisfaction with access to needed services.

#### 3.3.1 Model 1: total PASIPD-AR

The first SEM assessed the associations of demographic variables, SP-health, SP-fitness, and the total PASIPD-AR score on overall QoL. Model fit indices indicated acceptable approximation based on RMSEA (0.099, 90% CI: 0.075–0.124), with a satisfactory SRMR of 0.030. However, comparative fit indices were below ideal thresholds (CFI = 0.229, TLI = 0.904), indicating modest model parsimony.

All three WHOQOL-DIS-AR items loaded significantly onto the latent overall QoL construct, with item 2 (β = 0.901, *p* < 0.001) and item 1 (β = 0.773, *p* < 0.001) showing the greatest contributions, and item 26 (β = 0.357, *p* < 0.001) showing a moderate contribution (Table 4). The latent QoL factor explained 63.5% of the total variance (*R*^2^ = 0.635). In the structural model (Table 5), the strongest associations with overall QoL were observed for SP-health (β = 0.454, *p* < 0.001) and SP-fitness (β = 0.326, *p* < 0.001), representing large and moderate-to-large effect sizes, respectively, based on conventional benchmarks for standardized coefficients in behavioral sciences [e.g., β = 0.10 small, 0.30 medium, 0.50 large]. Sex was associated with a small but statistically significant difference in overall QoL (β = 0.151, *p* = 0.019), with females reporting higher scores. BMI demonstrated a small-to-moderate negative association with overall QoL (β = −0.205, *p* = 0.005), while the negative association with age was small and marginally significant (β = −0.134, *p* = 0.055). Critically, the total PASIPD-AR score showed a negligible effect (β = −0.005, *p* = 0.922). No other demographic variables were significant correlates in this model.

**Table 4:** Measurement model: standardized factor loadings for the latent overall QoL factor in both SEM models.

**WHOQOL-DIS-AR item**	**Model 1: Total PASIPD-AR**		**Model 2: PASIPD-AR factors**	
	**Std. Loading**	***R*** ^2^	**Std. Loading**	***R*** ^2^
Item 1: Physical health satisfaction	0.773	0.598	0.730	0.533
Item 2: Overall QoL perception	0.901	0.812	0.969	0.938
Item 26: Access to needed services	0.357	0.127	0.334	0.112

**Table 5:** Structural model: standardized associations and effect sizes with the latent overall QoL factor.

**Predictor**	**Model 1: Total PASIPD-AR**			**Model 2: PASIPD-AR Factors**		
	β	**p-value**	**ES**	β	**p-value**	**ES**
**Self-perception and demographics**						
SP-health	0.454	< 0.001	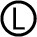	0.439	< 0.001	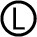
SP-fitness	0.326	< 0.001	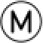	0.387	< 0.001	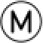
Sex (Female)	0.151	0.019	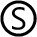	0.125	0.048	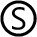
BMI	−0.205	0.005	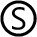	−0.155	0.053	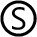
Age	−0.134	0.055	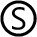	−0.105	0.101	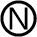
**Physical activity**						
Total PASIPD-AR Score	−0.005	0.922	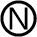	–	–	–
Home Repair Activities	–	–	–	0.074	0.246	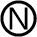
Household Activities	–	–	–	−0.114	0.081	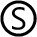
Sports and Recreational Activities	–	–	–	0.121	0.020	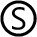
Occupational and Transportation Activities	–	–	–	−0.017	0.814	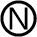
Other demographic variables	ns	>0.05	–	ns	>0.05	–

β, standardized regression coefficient; ES (Effect Size): 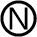 Negligible (|β| < 0.10), Small (0.10 ≤ |β| < 0.20), 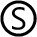 Medium (0.20 ≤ |β| < 0.50), 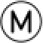 Large (|β| ≥ 0.50). Sex coded as 1—male, 2—female; positive β indicates higher QoL for females. “ns” denotes non-significant (p ≥ 0.10).

#### 3.3.2 Model 2: PASIPD-AR factors

The second SEM substituted the total PASIPD-AR score with the scores of its four domains: HRA, HHA, SRA, and OTA. The model fit was acceptable, with an RMSEA of 0.088 (90% CI: 0.065–0.110) and an SRMR of 0.029. While the chi-square test was significant [χ^2^_(30)_ = 82.69, *p* < 0.001], the TLI was adequate (0.937); however, the CFI remained low (0.373), likely due to the model's complexity and ordinal indicators.

All three WHOQOL-DIS-AR items loaded significantly onto the latent overall QoL construct (Table 4): item 2 (β = 0.969, *p* < 0.001), item 1 (β = 0.730, *p* < 0.001), and item 26 (β = 0.334, *p* < 0.001), confirming construct validity. The latent overall QoL factor explained 63.7% of the total variance (*R*^2^ = 0.637). The pattern of associations for demographic and self-perception variables remained consistent with Model 1 (Table 5). SP-health (β = 0.439, *p* < 0.001) and SP-fitness (β = 0.387, *p* < 0.001) continued to show large effect sizes. Among the four PASIPD-AR factors, only sports and recreational activities domain demonstrated a significant, though small, positive association with overall QoL (β = 0.121, *p* = 0.020). The associations for sex (β = 0.125, *p* = 0.048) and BMI (β = −0.155, *p* = 0.053) were also small in magnitude, with the link to BMI falling just outside conventional significance thresholds. The other PASIPD-AR factors (HRA, HHA, OTA) showed negligible to small effects that were not statistically significant. Detailed 95% confidence intervals for all estimates are provided in the Supplementary Table S5.

### 3.4 SEM: predictors of WHOQOL-DIS-AR components among IWPDs

A series of SEMs were conducted to provide a comprehensive overview of how demographic characteristics, SP-health and SP-fitness, and PA are associated with the various aspects of participants' QoL. Each SEM focused on a single latent construct that corresponded to one of the seven WHOQOL-DIS-AR components: four WHOQOL-BREF domains (Physical Health, Psychological, Social Relationships, and Environment), and three Disability module facets (Discrimination, Autonomy, and Inclusion/Participation). All models included a uniform set of correlates: age, gender, BMI, education level, occupation, type of disability, social relationships, income, use of disability aid, SP-health, SP-fitness, and either the total PASIPD-AR score (Model 1) or the four PASIPD-AR factors (HRA, HHA, SRA, and OTA; Model 2). All WHOQOL-DIS-AR components were treated as ordinal indicators, and the models were estimated using the DWLS method.

#### 3.4.1 Model 1: total PASIPD-AR

The fit indices for the seven SEMs constructed for the Arabic version of the WHO Quality of Life–Disabilities module components are presented in Table 6. All models exhibited good to excellent fit based on an RMSEA criterion of ≤ 0.12, supported by the SRMR and CFI in most cases. The Autonomy facet exhibited the best fit, as indicated by a non-significant chi-square test [χ^2^_(24)_ = 30.03, *p* = 0.184], low RMSEA (0.033), and excellent incremental fit indices (CFI = 0.987, TLI = 0.998, SRM*R* = 0.007). Similarly, the Discrimination facet demonstrated strong fit [χ^2^_(24)_ = 57.24, *p* < 0.001; RMSEA = 0.078; CFI = 0.946; SRM*R* = 0.017]. The Psychological domain exhibited good model fit [χ^2^_(69)_ = 160.24, *p* < 0.001; RMSEA = 0.076; CFI = 0.931; SRM*R* = 0.061]. The Social Relationships domain exhibited a moderately acceptable fit [χ^2^_(11)_ = 35.73, *p* < 0.001; RMSEA = 0.099; CFI = 0.864; SRMR < 0.001], with the RMSEA below the 0.12 threshold but the CFI slightly below the ideal value of 0.90. The Environmental [χ^2^_(104)_ = 461.65, *p* < 0.001; RMSEA = 0.123; CFI = 0.805] and Physical Health [χ^2^_(86)_ = 479.19, *p* < 0.001; RMSEA = 0.141; CFI = 0.667] domains exhibited RMSEA above the threshold, indicating less than optimum model approximation. However, the Environmental domain exhibited borderline model fit (RMSEA = 0.123), while the other models had acceptable TLI (>0.90), indicating an adequate relative fit.

**Table 6:** SEM fit indices for the latent WHOQOL-DIS-AR components.

**Component**	**χ^2^ (d*f*)**	***p***	**CFI**	**TLI**	**RMSEA (90 CI)**	**SRMR**	**Model fit interpretation**
Physical health	479.19 (86)	< 0.001	0.667	0.919	0.141 (0.129–0.153)	0.123	Poor fit
Psychological	160.24 (69)	< 0.001	0.931	0.985	0.076 (0.061–0.092)	0.061	Good fit
Social relationships	35.73 (11)	< 0.001	0.864	0.988	0.099 (0.065–0.135)	0.000	Acceptable fit
Environment	461.65 (104)	< 0.001	0.805	0.947	0.123 (0.111–0.136)	0.119	Borderline fit
Discrimination	57.24 (24)	< 0.001	0.946	0.993	0.078 (0.053–0.104)	0.017	Strong fit
Autonomy	30.03 (24)	0.184	0.987	0.998	0.033 (0.000–0.064)	0.007	Excellent fit
Inclusion/Participation	212.83 (69)	< 0.001	0.891	0.976	0.095 (0.081–0.109)	0.103	Acceptable

In conclusion, five of the seven WHOQOL-DIS-AR component-specific models met the established RMSEA threshold (< 0.12), demonstrating acceptable to good structural validity. The Physical Health and Environmental domains exhibited worse absolute fit, indicating potential model complexity or measurement constraints in these QoL components.

The factor loadings showed strong support for the validity of the latent QoL constructs. For the Physical Health domain, standardized factor loadings for the observed indicators ranged from 0.474 to 0.956, reflecting strong associations. The Psychological domain exhibited a similar pattern, with loadings ranging from 0.114 to 0.965, although one item exhibited a weak contribution. The Social Relationships domain had two items with high loadings (0.859 and 0.928), suggesting excellent internal consistency. The Environmental domain demonstrated moderate to strong loadings (0.434 to 0.931), despite slightly weaker overall fit. The loadings ranged from 0.700 to 1.026 for the Discrimination facet and from 0.739 to 0.887 for the Autonomy facet, both indicating strong convergent validity. Finally, the Inclusion/Participation facet showed substantial indicator contributions, with loadings ranging from 0.664 to 0.946, further affirming the robustness of the measurement structure within the SEM framework.

The models demonstrated several consistent and statistically significant patterns of association (Table 7). SP-fitness showed the strongest positive associations, with a large effect on the Psychological domain (β = 0.515) and medium effects on the Social Relationships (β = 0.456) and Environmental (β = 0.341) domains. SP-health also showed consistent positive associations of small-to-medium magnitude with the same domains. Notably, both SP-fitness and SP-health showed medium *negative* associations with the Physical Health domain and small-to-medium negative associations with the Inclusion/Participation facet. Other notable associations included a consistent pattern of small positive associations for education level with the Discrimination (β = 0.135), Autonomy (β = 0.140), and Inclusion/Participation (β = 0.236) facets. Occupation showed a medium negative association with Inclusion/Participation (β = −0.422) and small associations with several other domains. Social relationships demonstrated a medium positive association with the Autonomy facet (β = 0.265). The total PASIPD-AR score demonstrated a significant positive association of medium magnitude only with the Discrimination facet (β = 0.352), suggesting a specific link with perceptions of stigma, but showed negligible to small associations with other QoL components. Comprehensive 95% confidence intervals for all estimates are included in the Supplementary Table S7.

**Table 7:** Associations of demographics, SP-health and SP-fitness, and total PASIPD-AR score with WHOQOL-DIS-AR components.

**Predictor**	**Domains**				**Facets**		
	**Physical health**	**Psychological**	**Social relationships**	**Environmental**	**Discrimination**	**Autonomy**	**Inclusion/Participation**
	β **(ES)**	β **(ES)**	β **(ES)**	β **(ES)**	β **(ES)**	β **(ES)**	β **(ES)**
**Demographics**							
Age	↑0.208^***^ 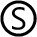	↓-0.266^***^ 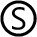	ns	ns	ns	↓−0.130^*^ 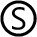	↑0.182^**^ 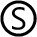
Sex (Female)	ns	ns	ns	ns	ns	↑0.107^*^ 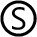	ns
BMI	ns	↓-0.122^*^ 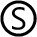	ns	↓-0.094^*^ 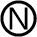	ns	ns	ns
Education level	↓-0.09 ^*^ 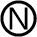	ns	↓-0.328^***^ 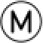	ns	↑0.135^*^ 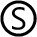	↑0.140^*^ 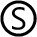	↑ 0.236^***^ 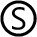
Occupation	↓-0.138^*^ 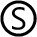	↑0.127^*^ 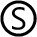	↑0.226^*^ 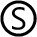	ns	↓-0.235^***^ 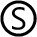	↑0.226^**^ 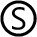	↓-0.422^***^ 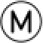
Type of disability	↑0.124^**^ 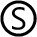	↓-0.146^***^ 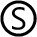	↑0.175^**^ 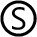	ns	ns	↓-0.140^*^ 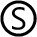	ns
Social relationships	↓-0.105^*^ 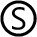	↑0.193^***^ 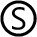	ns	ns	↓-0.162^**^ 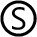	↑ 0.265^***^ 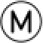	↓-0.091^*^ 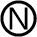
Income	NS	↑ 0.091^*^ 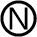	ns	ns	↓-0.218^***^ 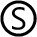	ns	↑ 0.070^*^ 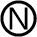
Disability aid	NS	↑0.103^*^ 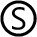	ns	ns	↓-0.129 ^**^ 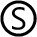	ns	↓-0.155^**^ 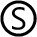
**Self-perception**							
SP-health	↓-0.310^***^ 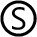	↑0.171^***^ 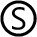	↑0.311^**^ 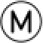	↑0.374^***^ 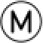	ns	↑0.172^**^ 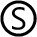	↓-0.224^***^ 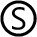
SP-fitness	↓-0.288^***^ 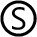	↑0.515^***^ 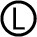	↑0.456^***^ 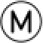	↑ 0.341^***^ 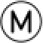	↑0.147^*^ 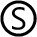	↑0.156^**^ 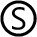	↓-0.365^***^ 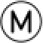
Total PASIPD-AR score	ns	↓-0.109^*^ 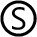	ns	↓-0.106^*^ 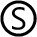	↑0.352^***^ 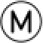	ns	ns

↑ or ↓ indicates the direction of the association. β, Standardized coefficient. ES (Effect Size): 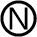 Negligible (|β| < 0.10), 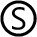 Small (0.10 ≤ |β| < 0.20), 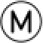 Medium (0.20 ≤ |β| < 0.50), 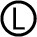 Large (|β| ≥ 0.50).

Significance: ^*^ p < 0.05; ^**^ p < 0.01; ^***^ p < 0.001.

“ns” denotes non-significant (p ≥ 0.05).

#### 3.4.2. Model 2: PASIPD-AR factors

Five of the seven models demonstrated an acceptable to excellent fit (Table 8). The Psychological and Social Relationships domains, as well as the Discrimination, Autonomy, and Inclusion/Participation facets, achieved satisfactory RMSEA and acceptable CFI and TLI. The Environmental domain exhibited borderline fit, while the Physical Health domain failed to meet key fit thresholds, indicating potential misspecification. Standardized factor loadings revealed strong convergent validity in several domains, with most items loading significantly and substantially onto their respective latent factors. Items such as item 17 (−0.979, Physical Health), item 25 (0.946, Environment), item 29 (1.010, Discrimination), and item 32 (0.889, Autonomy) demonstrated excellent indicator strength. Notably, several negatively phrased items exhibited strong negative loadings, consistent with the expectations of reverse scoring. Item 25 (Psychological domain) had a notably weak loading (0.075), suggesting a limited contribution in this context. For more detailed information regarding item-level contributions to each latent construct, the complete list of standardized factor loadings is presented in the Supplementary Table S1.

**Table 8:** SEM fit indices for WHOQOL-DIS-AR component models with PASIPD-AR factors as predictors.

**Component**	**χ^2^ (d*f*)**	**CFI**	**TLI**	**RMSEA (90% CI)**	**SRMR**	**Model fit interpretation**
Physical Health	479.19 (86)	0.583	0.916	0.132 [0.120, 0.143]	0.131	Poor fit
Psychological	160.24 (69)	0.886	0.980	0.078 [0.064, 0.091]	0.054	Acceptable fit
Social Relationships	31.38 (13)	0.889	0.992	0.079 [0.045, 0.113]	0.000	Acceptable fit
Environment	461.65 (104)	0.749	0.944	0.115 [0.104, 0.125]	0.122	Borderline/poor fit
Discrimination	11.28 (6)	0.917	0.992	0.076 [0.052, 0.099]	0.018	Good fit
Autonomy	1.63 (3)	0.988	0.999	0.025 [0.000, 0.058]	0.001	Excellent fit
Inclusion/Participation	47.58 (24)	0.849	0.973	0.090 [0.076, 0.103]	0.100	Acceptable

Multivariate SEM analyses revealed relationships among demographic characteristics, SP-health, SP-fitness, PASIPD-AR factors, and specific WHOQOL-DIS-AR components in IWPDs (Table 9). SP-health and SP-fitness showed the most consistent and substantial associations. Both variables showed medium-to-large positive associations with the Psychological (SP-health β = 0.198, Small; SP-fitness β = 0.479, Medium), Social Relationships (SP-health β = 0.295, Small; SP-fitness β = 0.340, Medium), and Environmental (SP-health β = 0.363, Medium; SP-fitness β = 0.308, Medium) domains. Conversely, they were associated with lower scores in the Physical Health domain (SP-health β = −0.325, Medium; SP-fitness β = −0.257, Small) and the Inclusion/Participation facet (SP-health β = −0.233, Small; SP-fitness β = −0.271, Small). Education level showed a consistent pattern of small positive associations with the facets of Discrimination (β = 0.141), Autonomy (β = 0.143), and Inclusion/Participation (β = 0.263). In contrast, occupation was associated with lower scores in the Physical Health domain (β = −0.124, Small) and the Discrimination (β = −0.194, Small) and Inclusion/Participation (β = −0.390, Medium) facets, while showing a small positive association with the Psychological domain (β = 0.149). The type of disability demonstrated a complex pattern, with a small positive association with the Physical Health domain (β = 0.118) but small negative associations with the Psychological domain (β = −0.165) and Autonomy facet (β = −0.129). Social relationships showed a small positive association with the Psychological domain (β = 0.174) and a medium positive association with the Autonomy facet (β = 0.261). The use of disability aids was associated with higher scores in the Psychological domain (β = 0.121, Small) and Autonomy facet (β = 0.154, Small), but with lower scores in the Discrimination (β = −0.144, Small) and Inclusion/Participation (β = −0.214, Small) facets.

**Table 9:** Associations of demographics, SP-health and SP-fitness, and PASIPD-AR factors with specific WHOQOL-DIS-AR components among IWPDs.

**Predictor**	**Domains**				**Facets**		
	**Physical health**	**Psychological**	**Social relationships**	**Environmental**	**Discrimination**	**Autonomy**	**Inclusion/participation**
	β **(ES)**	β **(ES)**	β **(ES)**	β **(ES)**	β **(ES)**	β **(ES)**	β **(ES)**
**Demographics**							
Age	↑ 0.193^***^ 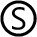	↓-0.261^***^ 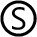	ns	ns	ns	↓-0.128^*^ 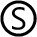	↑ 0.173^**^ 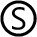
Sex (Female)	ns	↓−0.067^*^ 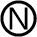	ns	ns	ns	↑0.114^*^ 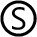	ns
BMI	ns	↓-0.111^*^ 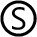	ns	↓-0.126^*^ 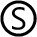	ns	ns	ns
Education level	ns	ns	↓-0.336^***^ 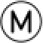	ns	↑0.141^*^ 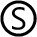	↑0.143^*^ 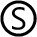	↑0.263^***^ 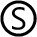
Occupation	↓−0.124^*^ 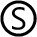	↑ 0.149^*^ 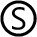	ns	ns	↓-0.194^**^ 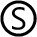	ns	↓-0.390^***^ 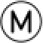
Type of disability	↑ 0.118^**^ 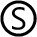	↓-0.165^***^ 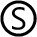	↑0.179^**^ 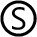	ns	ns	↓-0.129^*^ 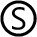	ns
Social relationships	ns	↑ 0.174^**^ 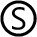	ns	ns	↓-0.152^*^ 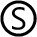	↑ (*β =* 0.261)^***^ 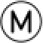	ns
Income	ns	↑0.111^*^ 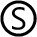	ns	ns	↓−0.199^**^ 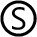	ns	↑0.077^*^ 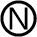
Disability aid	ns	↑0.121^*^ ( 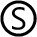	ns	ns	↓-0.144^*^ 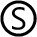	↑ (*β =* 0.154)^*^ 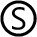	↓-0.214^***^ 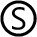
**Self-Perception**							
SP-health	↓−0.325^***^ 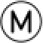	↑ 0.198^***^ 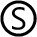	↑0.295^***^ 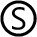	↑0.363^***^ 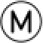	ns	↑0.188^**^ 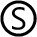	↓-0.233^***^ 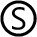
SP-fitness	↓−0.257^***^ 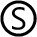	↑0.479^***^ 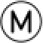	↑0.340^***^ 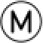	↑0.308^***^ 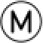	↑0.193^*^ 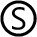	ns	↓-0.271^***^ 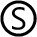
**PASIPD-AR Factors**							
Home Repair Activities	↑ 0.442^***^ 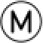	↓-0.377^***^ 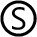	↓-0.446^***^ 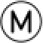	↓-0.259^**^ 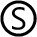	ns	↓-0.294^***^ 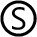	↑0.353^***^ 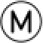
Household Activities	↓-0.336^***^ 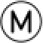	↑0.251^***^ 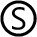	↑0.542^***^ 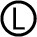	↑0.149^*^ 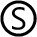	ns	↑0.531^***^ 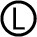	↓−0.399^***^ 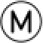
Sports and Recreational Activities	↓-0.087^*^ 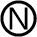	ns	ns	ns	↑0.288) ^***^ 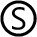	ns	ns
Occupational and Transportation Activities	↓-0.105^*^ 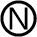	↓-0.080^*^ 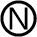	ns	↑0.081^*^ 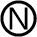	ns	↑0.025^*^ 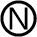	↓−0.060^*^ 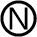

↑ or ↓ indicates the direction of the association. β = Standardized regression coefficient. ES (Effect Size): 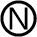 Negligible (|β| < 0.10), 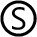 Small (0.10 ≤ |β| < 0.20), 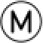 Medium (0.20 ≤ |β| < 0.50), 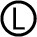 Large (|β| ≥ 0.50).

Significance: ns, non-significant. ^*^ p < 0.05; ^**^ p < 0.01; ^***^ p < 0.001.

Sex is coded as 1—Male, 2—Female. A positive β for Sex indicates a higher score for females.

The PASIPD-AR components showed distinct and nuanced associations. The home repair activities component was associated with higher scores in the Physical Health domain (β = 0.442, Medium) and Inclusion/Participation facet (β = 0.353, Medium), but with lower scores across multiple psychosocial domains: Psychological (β = −0.377, Medium), Social Relationships (β = −0.446, Medium), Environmental (β = −0.259, Small), and Autonomy (β = −0.294, Small). Conversely, the household activities showed a strong positive association with the Social Relationships domain (β = 0.542, Large) and Autonomy facet (β = 0.531, Large), and small-to-medium positive associations with the Psychological (β = 0.251) and Environmental (β = 0.149) domains, but was associated with lower scores in the Physical Health domain (β = −0.336, Medium) and Inclusion/Participation facet (β = −0.399, Medium). The sports and recreational activities domain demonstrated a small-to-medium positive association exclusively with the Discrimination facet (β = 0.288). The occupational and transportation activities domain showed only negligible associations across all components. Detailed 95% confidence intervals for all estimates are provided in the Supplementary Table S9.

## 4 Discussion

### 4.1 Psychometric properties and measurement considerations

This study aimed to examine self-assessed quality of life among individuals with physical disabilities in Saudi Arabia. It used the Arabic version of the WHO Quality of Life–Disabilities module questionnaire to assess overall QoL and its domains, and the PASIPD-AR to assess the types and amounts of physical activity. It then examined the relationships between Saudi IWPDs' demographics, SP-health and SP-fitness, use of mobility aids, and PA levels and their QoL.

Prior to discussing the substantive significance of the findings, it is essential to contextualize them within the psychometric features of the measurement model, particularly its cross-cultural application. The CFA revealed that the Physical Health and Environmental domains exhibited inadequate fit in our sample. This trend corresponds with the instrument's historical cultural adaptation. A significant adaptation of the WHOQOL-BRIEF was the exclusion of Item 21 relative to sexual satisfaction based on cultural sensitivity considerations. While culturally appropriate, this decision creates challenges for cross-cultural comparison and may omit an important QoL dimension, limiting direct cross-cultural comparability with studies using the full scale. Furthermore, ongoing difficulties with items like Item 14 (“opportunities for leisure”) were observed. Collectively, these findings indicate that the standard WHOQOL factor structure may not fully capture the conceptualization of these QoL dimensions within this specific cultural and disability context. Consequently, although descriptive and correlational results are provided for all domains, the findings for the Physical Health and Environmental domains should be regarded as preliminary and interpreted cautiously, as the latent variables for these constructs were not assessed with optimal reliability or direct international equivalence. This recurring measurement issue highlights the critical need for future research to develop more robust and culturally grounded assessments of quality of life for this population.

### 4.2 Quality of life levels and international comparisons

With this important caveat in mind, our findings revealed a mean total WHOQOL-DIS-AR score of 57.75 ± 24.24 on a 0–100 scale, indicating a moderate self-perception of QoL among Saudi IWPDs. However, substantial variability was observed across the WHOQOL-DIS-AR components. The highest mean score was observed for the Social Relationships domain (66.00 ± 26.76), followed by the Psychological domain (56.25 ± 21.58), and the lowest mean score was observed for the Environmental domain (46.00 ± 20.97), highlighting potential challenges in environmental aspects of daily life. Based on the equal-interval classification of total WHOQL-DIS-AR scores, 29.6% of participants reported a moderate level of QoL, 26.5% reported a good level of QoL, and 18.7% reported a very good level of QoL. In contrast, 15.7% reported a poor level of QoL, and 9.6% reported a very poor level of QoL. Overall, 54.8% of the participants rated their QoL from moderate to good, suggesting a generally favorable self-perception of QoL in this population.

Regrettably, no prior studies have comprehensively assessed overall QoL among IWPDs in Saudi Arabia using the WHOQOL-DIS-AR, limiting direct comparisons. Therefore, our study represents one of the first attempts to explore this topic within the Saudi context. However, Zahra et al. (2022) investigated the Psychological domain of QoL using the six relevant items from WHOQOL-BREF among 359 participants aged 15 years or older, including 32.3% with disabilities. Their findings showed significantly higher scores in the Psychological domain among participants without disabilities (mean = 68) than among those with disabilities (mean = 61, *p* < 0.01). Similarly, Algamdi and Al Amer (2024) assessed QoL among 369 adults in the city of Tabuk using the 36-Item Short Form Health Survey (SF-36), reporting a mean total SF-36 score of 69.5 out of 100, indicating a good level of perceived wellbeing.

While these instruments differ in scope and conceptual focus—the SF-36 emphasizes health functioning and the WHOQOL-DIS focuses on subjective wellbeing and social participation—our findings suggest an association between IWPDs status and somewhat lower levels of QoL compared to the general population. This difference is further supported by Madhesh (2023), who conducted qualitative interviews with 11 students with disabilities at Shaqra University. He examined QoL across several domains, including personal development, self-determination, interpersonal relations, social inclusion, rights, emotional wellbeing, and material wellbeing. His findings indicated a notably low level of QoL among these students, which was associated with institutional barriers, inadequate accommodation, and limited awareness among faculty and peers. Similarly, a study in Oman involving 35 students with various impairments reported relatively high levels of satisfaction in social relationships (mean = 72.5), environment (mean = 70.0), and psychological health (mean = 68.25), and moderate satisfaction in physical health (mean = 56.75), based on the WHOQOL-BREF (Hilal Al-Mamari et al., 2025).

The evidence from studies conducted in other countries indicates that while individuals with disabilities report varied, but predominantly moderate, overall quality of life, these observations align with the results of our study in Saudi Arabia. Zheng et al. (2014) conducted a comprehensive investigation involving 1,853 IWPDs in China (60.7% male), reporting mean QoL scores of 41.25–55.5, indicating moderate overall QoL, even among those with comparatively slight impairments. The international WHOQOL-DIS field trial conducted in 12 countries indicated that individuals with disabilities consistently reported an inferior QoL compared to the general population; nonetheless, the instrument exhibited robust cross-cultural validity and effectively differentiated between varying degrees of disability severity (Power et al., 2010). Balzer-Geldsetzer et al. (2018) discovered that individuals with Parkinson's disease exhibited markedly lower QoL, which was associated with increased disease severity, prolonged illness duration, and depressed symptoms. In a comparative study, Valeikiene et al. (2008) observed no significant disparities in overall QoL among patients with Parkinson's disease, osteoarthritis, and healthy controls; however, the patients with Parkinson's disease exhibited markedly lower scores in mobility, daily functioning, and work capacity, especially within the Independence domain of the WHOQOL-100.

In a study in India, Kuvalekar et al. (2015) evaluated the QoL of 130 IWPDs using the WHOQOL-BREF, revealing the lowest scores in the Psychological domain, indicative of difficulties associated with negative emotions, body image, spirituality, and self-esteem. In contrast, a study assessing health-related QoL among post-paralytic Polio survivors in Nigeria reported a mean QoL score for overall QoL and overall health items of 3.3 ± 0.8 and 3.6 ± 0.7, respectively (Kaka et al., 2011). The highest mean score was in the Psychological Health domain (54.8 ± 11.4), while the lowest score was in the Social Relationships domain (41.3 ±10.9). Collectively, these data underscore global uniformity in moderate QoL levels among individuals with disabilities and highlight associations between cultural, health-related, and structural factors and QoL outcomes.

### 4.3 Key correlates of quality of life

Although numerous studies have examined the determinants of quality of life in the general population, relatively little research has focused on IWPDs, particularly within the Saudi Arabian context. Understanding the specific factors that are associated with QoL in this population is essential for designing effective, targeted interventions. Our study employed SEM to assess the demographic, psychosocial, and physical activity factors associated with both overall QoL and its domains, as measured by the Arabic version of the WHO Quality of Life–Disabilities module.

Our findings revealed that among individuals with physical disabilities, quality of life was associated with a multifaceted set of factors, with SP-health and SP-fitness emerging as the most robust and consistent contributors across domains. Sex, BMI, education level, occupation, and domain-specific physical activity also demonstrated meaningful associations with various QoL components, underscoring the multidimensional nature of wellbeing in this population. It is crucial to emphasize that these relationships, derived from a cross-sectional design, are associational and do not support causal inference, as temporal precedence and the exclusion of confounding cannot be established (Kesmodel, 2018). Longitudinal and interventional studies are necessary to determine whether modifying these factors leads to measurable improvements in QoL.

Interpreting the data using the social model of disability adds vital context. This model, a cornerstone of modern disability studies, posits that disability arises from the interaction between individuals with impairment and disabling social, attitudinal, and environmental barriers, rather than from the impairment itself (Oliver, 2013). Within this framework, the associations with education, employment, and income can be viewed as manifestations of structural inequities that systematically limit participation and opportunity (Haegele and Hodge, 2016). The complex role of household activities may also reflect the additional physical and emotional labor required to navigate non-inclusive environments. Thus, while our findings identify individual-level correlates, they also point to underlying structural determinants within the Saudi context that warrant further investigation as primary targets for policy and intervention.

A notable finding was the sex-based difference in overall QoL, with females reporting higher levels than males. This pattern aligns with studies suggesting that women with disabilities often benefit from stronger social networks and adaptive coping strategies (Lone et al., 2024), although other evidence indicates that men experience higher QoL through greater social mobility and engagement (Hamadneh and Almogbel, 2023). Such discrepancies may stem from sociocultural norms and gender-role expectations. In particular, men with disabilities may experience internal conflicts between societal ideals of masculinity and independence, which could adversely affect psychological wellbeing (Lone et al., 2024).

Age showed a modest negative association with overall QoL, suggesting a slight decline in perceived wellbeing with advancing age. This finding aligns with prior research linking aging to reductions in physical and psychological functioning and engagement in daily activities (Yang et al., 2018; Buchholz and Janssen, 2023). SP-health and SP-fitness were the most consistent and powerful correlates of QoL across domains, confirming perceived health as a central determinant of subjective wellbeing (Helliwell et al., 2020; Maniscalco et al., 2020). The association is likely bidirectional—better perceived health may both promote and reflect higher QoL—but the present cross-sectional design limits any causal inference.

Education level was a significant positive correlate of Autonomy, Inclusion/Participation, and Discrimination facets, consistent with previous studies linking higher education to greater resilience, coping capacity, and life satisfaction among individuals with disabilities (Alhumaid et al., 2024c; Kuvalekar et al., 2015; Jani et al., 2022; Addabbo et al., 2016; Ferrer-i-Carbonell, 2005). From a social model perspective, this association can be interpreted not merely as an individual achievement but as a reflection of the structural and societal advantages that education confers, such as greater access to economic resources, social capital, and assistive technologies, which in turn facilitate autonomy and social participation (Oliver, 2013). Occupational status also demonstrated mixed associations—positively with Autonomy but negatively with Physical Health and Discrimination. This pattern is indicative of persistent systemic workplace barriers, including inadequate accessibility, a lack of reasonable accommodation, and prevailing stigmatizing attitudes that can negate the potential benefits of employment and even pose risks to wellbeing (Kuvalekar et al., 2015; Winters, 2011; Albrecht and Devlieger, 1999).

Social relationships were associated with greater psychological wellbeing and autonomy, consistent with prior research showing that supportive networks enhance emotional resilience and adaptive functioning (Tough et al., 2017; Kapp, 2018). However, unmeasured factors such as family cohesion or personality traits may have influenced these associations (Dehghankar et al., 2024; Peleg and Peleg, 2025; Flores-Buils and Andrés-Roqueta, 2022). The use of assistive devices showed a dual pattern—positively associated with Psychological QoL but negatively with Inclusion/Participation and Discrimination facets—illustrating how assistive technologies can simultaneously empower individuals and reinforce visibility or stigma (Nyame et al., 2024; Mahmoudi-Dehaki et al., 2025).

Finally, income emerged as a significant structural correlate of QoL, especially within the Environmental domain. In alignment with prior studies, financial stability improves access to healthcare, assistive devices, and social participation, while financial insecurity limits engagement and heightens vulnerability (Jaffar et al., 2025; Sabri et al., 2023; Huang et al., 2020; Haque et al., 2024). Nevertheless, the strength of this association is likely context-dependent and may vary across different socioeconomic and geographic settings (Schwartz et al., 2019; Edwards et al., 2023; Santhalingam et al., 2022; Cai et al., 2025). Future research should utilize longitudinal, stratified, or multilevel methodologies to clarify the direct and indirect impacts of income on QoL outcomes.

### 4.4. The complex relationship between physical activity and quality of life

Interestingly, while the total PASIPD-AR score was not a significant correlate of overall quality of life, its domains demonstrated differential associations. This finding supports Martin Ginis (Martin Ginis, 2025), who argued that the effects of physical activity on QoL in individuals with disabilities are often underestimated due to conceptual and measurement inconsistencies. In our study, the HHA domain was positively associated with the Psychological domain and Autonomy facet of QoL, while the HRA domain was negatively associated with the Psychological and Social Relationships domains of QoL. These findings suggest that the type and context of PA are critical; some forms of activity may foster engagement and purpose, while others may be physically demanding or stressful, potentially diminishing wellbeing. Jacob et al. (2023) and Carbó-Carreté et al. (2016) reached similar conclusions, emphasizing the role of perceived PA and individualized support in associations with higher QoL among adults with intellectual disabilities. Additionally, findings from an intervention study (Diz et al., 2021) suggest that structured PA programs are associated with improvements in adaptive behavior, motor skills, and social outcomes in adults with intellectual disability.

A complex pattern was identified for household activities, indicating a positive correlation with psychological wellbeing and a negative correlation with physical health in multivariate models. This divergence requires careful interpretation based on methodological and contextual factors. The correlation matrix indicates that the negative coefficient for HHA in the physical health model may reflect a statistical suppression effect. The bivariate association between HHA and physical health was positive (*r* = 0.39); however, this association reversed in sign when other highly correlated activity domains, such as home repair activities domain (*r* = 0.80) and sports and recreational activities (*r* = 0.64), were considered. This reversal corresponds with suppression, wherein the shared variance among predictors alters observed relationships without reflecting a true negative association (MacKinnon et al., 2000). The multivariate coefficient appears to represent a statistical artifact instead of a significant inverse relationship.

This finding's interpretation is improved by considering the context. The literature on occupational science indicates that HHA may qualitatively differ from discretionary participation, often serving as obligatory tasks essential for role maintenance and daily functioning (Hammell, 2004; Wilcock, 2006). In contexts of disability, these activities may pertain to perceived competence and physical strain, shaped by environmental accessibility and social support. Empirical evidence from Saudi Arabia underscores the complexity of this issue, demonstrating that environmental barriers and limited accessibility elevate the perceived demands of daily activities for individuals with disabilities (Al-Hazzaa, 2018; Albujulaya and Stevinson, 2025). The differing associations of household activities across QoL domains suggest both statistical and contextual dimensions. The results suggest that the quality of experience and environmental factors linked to daily activities may influence the relationship between participation and different aspects of quality of life. Following the guidance of Hurlbert et al. (2019), it is crucial to interpret these findings by considering effect sizes, conceptual plausibility, and contextual coherence, rather than depending exclusively on statistical significance.

### 4.5 Limitations and future directions

This study presents several significant limitations that must be acknowledged in the interpretation of the findings. First, the cross-sectional design limits causal inference and constrains the generalizability of findings over time or across diverse populations. Longitudinal studies are crucial for evaluating temporal changes and the consistency of these relationships (Savitz and Wellenius, 2023). Furthermore, we performed several statistical tests across seven QoL domains without implementing a universal alpha correction, which may enhance sensitivity to theoretically relevant associations but also elevates the risk of Type I errors. Consequently, findings that are weaker or less consistent warrant careful interpretation.

Second, significant psychometric limitations were observed regarding the measurement of essential constructs within WHOQOL-BREF. The CFA indicated that the Physical Health and Environmental domains demonstrated poor fit (CFI < 0.90, RMSEA > 0.12), highlighting specific concerns such as items with low explained variance (e.g., Item 3) and insufficient convergent validity (AVE < 0.50). This pattern corresponds with challenges noted during the cultural adaptation of the instrument, including the prior exclusion of a sensitive item and persistent issues with other items, such as Item 14 (Alhumaid et al., 2024c). As a result, the latent variable scores for these domains likely exhibit significant measurement error, which may bias their relationships with other variables. This is especially pertinent to the intricate relationship between household activity and physical health, which should be considered highly preliminary. Future research should focus on qualitative or mixed methods approaches to clarify the conceptualization of these domains within this context, which may result in a more precise and valid measurement model.

Third, the generalizability of our findings is limited by the characteristics of the sample. The majority of participants were unemployed (80.4%) and low-income (71.3% earning below 5,000 SAR monthly), indicating that our findings may be particularly relevant to economically disadvantaged IWPDs. Furthermore, recruitment through disability organizations may have introduced selection bias by excluding more isolated individuals who might demonstrate different quality of life outcomes. Future research must incorporate community-based outreach to recruit a more diverse sample and validate these findings across different socioeconomic strata. The current analysis regarded the sample as a single homogeneous group and did not perform subgroup analyses based on significant characteristics, including type of disability, gender, or geographic region. The relationships among physical activity, psychosocial factors, and quality of life may vary significantly across these subgroups. The barriers encountered by individuals with polio may differ from those experienced by individuals with paraplegia. The statistical power of our study was insufficient to thoroughly investigate these potential disparities. Subsequent studies utilizing larger, intentionally selected cohorts should explore these nuances to enhance the development of more targeted and effective interventions.

Fourth, several significant psychosocial and environmental factors were not analyzed, including levels of stress and depression, perceived social or family support, and access to healthcare. These variables may be linked to QoL and should be examined in future research to improve understanding, particularly concerning age-related differences in social support and psychological wellbeing among individuals with disabilities (Murphy et al., 2023).

Fifth, this study did not gather data on the onset or duration of participants' disabilities. The length of an individual's experience with physical disability may significantly influence their psychological adjustment and perceived QoL (Alhumaid et al., 2024c). Future research should examine the relationships between the duration of disability and adaptation processes, along with the long-term effects on wellbeing. Furthermore, future research should use participatory and emancipatory research methodologies, in accordance with disability studies frameworks, to ensure that IWPDs' interests and lived experiences directly shape the research agenda focused on removing structural barriers.

Finally, our dependence on self-reported measures may introduce recall and social desirability biases. Future studies should incorporate objective tools like accelerometers for physical activity (Dyrstad et al., 2014), bioimpedance or DXA for body composition (Toombs et al., 2012), and clinical assessments for health and fitness (Rikli and Jones, 2013), to improve accuracy and validity of findings.

## 5 Conclusions

This study identified several key factors linked to the quality of life among individuals with physical disabilities in Saudi Arabia, highlighting possible pathways for supportive interventions. Self-perceived health and fitness demonstrated the most reliable connections across various QoL domains, underscoring the importance of comprehensive strategies that consider both physical and mental wellbeing. However, these relationships should be interpreted with caution, as they rely on self-reported measures and a cross-sectional design.

Socioeconomic factors, especially education and income, were associated with autonomy, social inclusion, and experiences of discrimination, emphasizing the significance of policies that improve educational access and economic empowerment.

Physical activity demonstrated complex, domain-specific connections with quality of life. Although the overall activity scores did not correlate with total QoL, specific patterns related to different domains were observed. For instance, household activity demonstrated positive correlations in bivariate analyses, yet revealed negative associations in multivariate models, highlighting intricate, context-dependent effects. These results—particularly regarding the physical health domain—should be interpreted cautiously due to possible measurement limitations. The findings indicate that customized activity programs highlighting meaningful and contextually relevant activities could be more effective than focusing solely on overall activity volume.

Gender differences, with males reporting lower QoL than females, suggest the possible advantages of implementing gender-sensitive support strategies. Future research should investigate the underlying causes of these differences through targeted subgroup analyses.

In conclusion, these correlational findings suggest promising directions for enhancing QoL; however, future intervention studies are essential to determine causal relationships and confirm whether modifying these factors produces tangible improvements. The extensive framework proposed by these associations—covering healthcare, socioeconomic barriers, customized activities, and psychosocial support—should be considered a preliminary model to be validated through future experimental and longitudinal research.

## Data Availability

The raw data supporting the conclusions of this article will be made available by the authors, without undue reservation.
