# Supplementary material for: Moving toward wellbeing: physical activity and quality of life in individuals with physical disabilities in Saudi Arabia
Source: Front Psychol. 2025 Nov 3;16:1684083. doi: 10.3389/fpsyg.2025.1684083 (PMC12620481; doi:10.3389/fpsyg.2025.1684083)

Results

Confirmatory Factor Analysis

Model fit

Chi-square test

Model	X^2	df	p
Baseline model	34715.831	595	
Factor model	1976.430	539	< .001

Note. The estimator is DWLS. The test statistic is scaled.shifted. The standard error method is robust.sem.

Additional fit measures

Fit indices

Index	Value
Comparative Fit Index (CFI)	0.958
Tucker-Lewis Index (TLI)	0.953
Bentler-Bonett Non-normed Fit Index (NNFI)	0.953
Bentler-Bonett Normed Fit Index (NFI)	0.943
Parsimony Normed Fit Index (PNFI)	0.890
Bollen's Relative Fit Index (RFI)	0.937
Bollen's Incremental Fit Index (IFI)	0.958
Relative Noncentrality Index (RNI)	0.958

Note. Except for the PNFI, the fit indices are scaled because of categorical variables in the data.

Information criteria

Information criteria are only available with ML-type estimators

[illegible]	[illegible]
Akaike (AIC)	
Bayesian (BIC)	
Sample-size adjusted Bayesian (SSABIC)	

Other fit measures

Metric	Value
Root mean square error of approximation (RMSEA)	0.108
RMSEA 90% CI lower bound	0.103
RMSEA 90% CI upper bound	0.113
RMSEA p-value	4.621×10^{-13}
Standardized root mean square residual (SRMR)	0.117
Hoelter's critical N (α = .05)	46.764
Hoelter's critical N (α = .01)	48.628
Goodness of fit index (GFI)	0.983
McDonald fit index (MFI)	0.005
Expected cross validation index (ECVI)	

Note. The RMSEA results are scaled because of categorical variables in the data.

R-Squared

	R^2
WHOQOL_3	0.160
WHOQOL_4	0.605
WHOQOL_10	0.457
WHOQOL_15	0.752
WHOQOL_16	0.371
WHOQOL_17	0.873
WHOQOL_18	0.639
WHOQOL_5	0.906
WHOQOL_6	0.768
WHOQOL_7	0.257
WHOQOL_11	0.302
WHOQOL_19	0.951
WHOQOL_26	0.890
WHOQOL_20	0.730
WHOQOL_22	0.751
WHOQOL_8	0.225
WHOQOL_9	0.539
WHOQOL_12	0.414
WHOQOL_13	0.555
WHOQOL_14	0.489
WHOQOL_23	0.606
WHOQOL_24	0.122
WHOQOL_25	0.620
WHOQOL_28	0.485
WHOQOL_29	0.515
WHOQOL_30	0.971
WHOQOL_31	0.904
WHOQOL_32	0.711
WHOQOL_33	0.870
WHOQOL_34	0.953
WHOQOL_35	0.950
WHOQOL_36	0.887
WHOQOL_37	0.980
WHOQOL_38	0.979
WHOQOL_39	0.914

Parameter estimates

Factor loadings

						95% Confidence Interval	
Factor	Indicator	Estimate	Std. Error	z-value	p	Lower	Upper
Factor 1	WHOQOL_3	1.000	0.000			1.000	1.000
	WHOQOL_4	1.944	0.249	7.794	< .001	1.455	2.433
	WHOQOL_10	1.689	0.241	7.023	< .001	1.218	2.161
	WHOQOL_15	2.167	0.267	8.117	< .001	1.644	2.690
	WHOQOL_16	1.522	0.221	6.887	< .001	1.089	1.956
	WHOQOL_17	2.335	0.290	8.041	< .001	1.766	2.904
	WHOQOL_18	1.997	0.267	7.475	< .001	1.474	2.521
Factor 2	WHOQOL_5	1.000	0.000			1.000	1.000
	WHOQOL_6	0.921	0.023	39.777	< .001	0.875	0.966
	WHOQOL_7	0.533	0.056	9.505	< .001	0.423	0.643
	WHOQOL_11	0.577	0.048	12.056	< .001	0.483	0.671
	WHOQOL_19	1.024	0.020	50.463	< .001	0.985	1.064
	WHOQOL_26	0.991	0.020	50.231	< .001	0.952	1.029
Factor 3	WHOQOL_20	1.000	0.000			1.000	1.000
	WHOQOL_22	1.014	0.033	30.538	< .001	0.949	1.079
Factor 4	WHOQOL_8	1.000	0.000			1.000	1.000
	WHOQOL_9	1.547	0.175	8.839	< .001	1.204	1.890
	WHOQOL_12	1.357	0.156	8.716	< .001	1.052	1.662
	WHOQOL_13	1.570	0.180	8.732	< .001	1.218	1.922
	WHOQOL_14	1.473	0.175	8.424	< .001	1.130	1.816
	WHOQOL_23	1.641	0.180	9.129	< .001	1.289	1.993
	WHOQOL_24	0.736	0.149	4.954	< .001	0.445	1.028
	WHOQOL_25	1.659	0.184	9.040	< .001	1.299	2.019
Factor 5	WHOQOL_28	1.000	0.000			1.000	1.000
	WHOQOL_29	1.030	0.076	13.538	< .001	0.881	1.179
	WHOQOL_30	1.414	0.089	15.910	< .001	1.240	1.588
Factor 6	WHOQOL_31	1.000	0.000			1.000	1.000
	WHOQOL_32	0.887	0.029	30.838	< .001	0.831	0.943
	WHOQOL_33	0.981	0.029	33.473	< .001	0.924	1.039
Factor 7	WHOQOL_34	1.000	0.000			1.000	1.000
	WHOQOL_35	0.999	0.018	55.711	< .001	0.963	1.034
	WHOQOL_36	0.965	0.020	47.712	< .001	0.925	1.005
	WHOQOL_37	1.014	0.030	33.313	< .001	0.955	1.074
	WHOQOL_38	1.014	0.018	56.571	< .001	0.978	1.049
	WHOQOL_39	0.980	0.022	44.675	< .001	0.937	1.023

Factor variances

					95% Confidence Interval	
Factor	Estimate	Std. Error	z-value	p	Lower	Upper
Factor 1	0.160	0.040	4.008	< .001	0.082	0.239
Factor 2	0.906	0.031	28.933	< .001	0.845	0.968
Factor 3	0.730	0.041	17.790	< .001	0.650	0.811
Factor 4	0.225	0.049	4.598	< .001	0.129	0.321
Factor 5	0.485	0.053	9.102	< .001	0.381	0.590
Factor 6	0.904	0.030	29.805	< .001	0.844	0.963
Factor 7	0.953	0.029	32.969	< .001	0.896	1.009

Factor Covariances

							95% Confidence Interval	
			Estimate	Std. Error	z-value	p	Lower	Upper
Factor 1	↔	Factor 2	0.281	0.038	7.383	< .001	0.207	0.356
Factor 1	↔	Factor 3	0.268	0.036	7.508	< .001	0.198	0.339
Factor 1	↔	Factor 4	0.160	0.027	5.852	< .001	0.107	0.214
Factor 1	↔	Factor 5	−0.067	0.021	−3.225	0.001	−0.107	−0.026
Factor 1	↔	Factor 6	−0.070	0.025	−2.825	0.005	−0.118	−0.021
Factor 1	↔	Factor 7	0.219	0.032	6.804	< .001	0.156	0.282
Factor 2	↔	Factor 3	0.680	0.033	20.819	< .001	0.616	0.744
Factor 2	↔	Factor 4	0.403	0.044	9.066	< .001	0.316	0.491
Factor 2	↔	Factor 5	−0.125	0.048	−2.582	0.010	−0.220	−0.030
Factor 2	↔	Factor 6	−0.146	0.058	−2.514	0.012	−0.259	−0.032
Factor 2	↔	Factor 7	0.366	0.039	9.374	< .001	0.290	0.443
Factor 3	↔	Factor 4	0.316	0.037	8.433	< .001	0.242	0.389
Factor 3	↔	Factor 5	−0.183	0.048	−3.846	< .001	−0.276	−0.090
Factor 3	↔	Factor 6	−0.199	0.057	−3.507	< .001	−0.310	−0.088
Factor 3	↔	Factor 7	0.389	0.039	9.987	< .001	0.313	0.466
Factor 4	↔	Factor 5	−0.057	0.025	−2.243	0.025	−0.106	−0.007
Factor 4	↔	Factor 6	−0.075	0.030	−2.493	0.013	−0.134	−0.016
Factor 4	↔	Factor 7	0.231	0.033	7.078	< .001	0.167	0.295
Factor 5	↔	Factor 6	0.537	0.038	14.069	< .001	0.462	0.612
Factor 5	↔	Factor 7	−0.135	0.038	−3.540	< .001	−0.209	−0.060
Factor 6	↔	Factor 7	−0.113	0.049	−2.291	0.022	−0.209	−0.016

Residual variances

					95% Confidence Interval	
Indicator	Estimate	Std. Error	z-value	p	Lower	Upper
WHOQOL_3	0.840	0.000			0.840	0.840
WHOQOL_4	0.395	0.000			0.395	0.395
WHOQOL_10	0.543	0.000			0.543	0.543
WHOQOL_15	0.248	0.000			0.248	0.248
WHOQOL_16	0.629	0.000			0.629	0.629
WHOQOL_17	0.127	0.000			0.127	0.127
WHOQOL_18	0.361	0.000			0.361	0.361
WHOQOL_5	0.094	0.000			0.094	0.094
WHOQOL_6	0.232	0.000			0.232	0.232
WHOQOL_7	0.743	0.000			0.743	0.743
WHOQOL_11	0.698	0.000			0.698	0.698
WHOQOL_19	0.049	0.000			0.049	0.049
WHOQOL_26	0.110	0.000			0.110	0.110
WHOQOL_20	0.270	0.000			0.270	0.270
WHOQOL_22	0.249	0.000			0.249	0.249
WHOQOL_8	0.775	0.000			0.775	0.775
WHOQOL_9	0.461	0.000			0.461	0.461
WHOQOL_12	0.586	0.000			0.586	0.586
WHOQOL_13	0.445	0.000			0.445	0.445
WHOQOL_14	0.511	0.000			0.511	0.511
WHOQOL_23	0.394	0.000			0.394	0.394
WHOQOL_24	0.878	0.000			0.878	0.878
WHOQOL_25	0.380	0.000			0.380	0.380
WHOQOL_28	0.515	0.000			0.515	0.515
WHOQOL_29	0.485	0.000			0.485	0.485
WHOQOL_30	0.029	0.000			0.029	0.029
WHOQOL_31	0.096	0.000			0.096	0.096
WHOQOL_32	0.289	0.000			0.289	0.289
WHOQOL_33	0.130	0.000			0.130	0.130
WHOQOL_34	0.047	0.000			0.047	0.047
WHOQOL_35	0.050	0.000			0.050	0.050
WHOQOL_36	0.113	0.000			0.113	0.113
WHOQOL_37	0.020	0.000			0.020	0.020
WHOQOL_38	0.021	0.000			0.021	0.021
WHOQOL_39	0.086	0.000			0.086	0.086

Thresholds

						95% Confidence Interval	
Indicator	Threshold	Estimate	Std. Error	z-value	p	Lower	Upper
WHOQOL_3	t1	−0.811	0.094	−8.671	0.000	−0.994	−0.628
	t2	−0.310	0.084	−3.679	2.345×10^{-4}	−0.475	−0.145
	t3	0.209	0.083	2.498	0.012	0.045	0.372
	t4	0.842	0.094	8.914	0.000	0.657	1.027
WHOQOL_4	t1	−0.588	0.088	−6.664	2.669×10^{-11}	−0.761	−0.415
	t2	−0.120	0.083	−1.447	0.148	−0.283	0.043
	t3	0.209	0.083	2.498	0.012	0.045	0.372
	t4	0.922	0.097	9.508	0.000	0.732	1.112
WHOQOL_10	t1	−1.233	0.110	−11.180	0.000	−1.450	−1.017
	t2	−0.842	0.094	−8.914	0.000	−1.027	−0.657
	t3	−0.537	0.087	−6.150	7.753×10^{-10}	−0.708	−0.366
	t4	0.842	0.094	8.914	0.000	0.657	1.027
WHOQOL_15	t1	−0.500	0.087	−5.763	8.283×10^{-9}	−0.669	−0.330
	t2	−0.253	0.084	−3.023	0.002	−0.418	−0.089
	t3	0.186	0.083	2.236	0.025	0.023	0.350
	t4	1.307	0.114	11.421	0.000	1.082	1.531
WHOQOL_16	t1	−1.417	0.121	−11.672	0.000	−1.655	−1.179
	t2	−1.065	0.102	−10.402	0.000	−1.265	−0.864
	t3	−0.368	0.085	−4.332	1.475×10^{-5}	−0.534	−0.201
	t4	1.084	0.103	10.508	0.000	0.882	1.286
WHOQOL_17	t1	−0.889	0.096	−9.273	0.000	−1.077	−0.701
	t2	−0.287	0.084	−3.417	6.338×10^{-4}	−0.452	−0.122
	t3	0.065	0.083	0.789	0.430	−0.097	0.228
	t4	1.307	0.114	11.421	0.000	1.082	1.531
WHOQOL_18	t1	−1.188	0.108	−11.002	0.000	−1.400	−0.976
	t2	−0.668	0.090	−7.427	1.108×10^{-13}	−0.844	−0.491
	t3	−0.120	0.083	−1.447	0.148	−0.283	0.043
	t4	1.188	0.108	11.002	0.000	0.976	1.400
WHOQOL_5	t1	−0.939	0.098	−9.624	0.000	−1.130	−0.748
	t2	−0.231	0.084	−2.761	0.006	−0.395	−0.067
	t3	0.695	0.091	7.680	1.599×10^{-14}	0.518	0.873
	t4	1.307	0.114	11.421	0.000	1.082	1.531
WHOQOL_6	t1	−1.360	0.118	−11.559	0.000	−1.590	−1.129
	t2	−0.524	0.087	−6.021	1.734×10^{-9}	−0.695	−0.354
	t3	0.186	0.083	2.236	0.025	0.023	0.350
	t4	0.889	0.096	9.273	0.000	0.701	1.077
WHOQOL_7	t1	−1.585	0.134	−11.803	0.000	−1.848	−1.322
	t2	−0.403	0.085	−4.724	2.316×10^{-6}	−0.570	−0.236
	t3	0.011	0.083	0.132	0.895	−0.151	0.173
	t4	1.307	0.114	11.421	0.000	1.082	1.531
WHOQOL_11	t1	−1.084	0.103	−10.508	0.000	−1.286	−0.882
	t2	−0.220	0.084	−2.630	0.009	−0.383	−0.056
	t3	0.811	0.094	8.671	0.000	0.628	0.994
	t4	1.479	0.126	11.756	0.000	1.232	1.726
WHOQOL_19	t1	−1.233	0.110	−11.180	0.000	−1.450	−1.017
	t2	−0.873	0.095	−9.154	0.000	−1.060	−0.686
	t3	−0.562	0.088	−6.407	1.482×10^{-10}	−0.734	−0.390
	t4	0.723	0.091	7.930	2.220×10^{-15}	0.544	0.902
WHOQOL_26	t1	−1.479	0.126	−11.756	0.000	−1.726	−1.232
	t2	−0.991	0.099	−9.965	0.000	−1.186	−0.796
	t3	−0.500	0.087	−5.763	8.283×10^{-9}	−0.669	−0.330
	t4	0.752	0.092	8.179	2.220×10^{-16}	0.572	0.932
WHOQOL_20	t1	−1.333	0.116	−11.493	0.000	−1.560	−1.105
	t2	−0.873	0.095	−9.154	0.000	−1.060	−0.686
	t3	−0.668	0.090	−7.427	1.108×10^{-13}	−0.844	−0.491
	t4	0.723	0.091	7.930	2.220×10^{-15}	0.544	0.902
WHOQOL_22	t1	−0.991	0.099	−9.965	0.000	−1.186	−0.796
	t2	−0.781	0.093	−8.426	0.000	−0.963	−0.599
	t3	−0.065	0.083	−0.789	0.430	−0.228	0.097
	t4	0.796	0.093	8.549	0.000	0.613	0.978
WHOQOL_8	t1	−0.253	0.084	−3.023	0.002	−0.418	−0.089
	t2	0.857	0.095	9.034	0.000	0.671	1.043
	t3	1.447	0.124	11.718	0.000	1.205	1.689
	t4	2.225	0.223	9.962	0.000	1.787	2.663
WHOQOL_9	t1	−0.681	0.090	−7.554	4.241×10^{-14}	−0.858	−0.505
	t2	−0.463	0.086	−5.374	7.704×10^{-8}	−0.632	−0.294
	t3	−0.120	0.083	−1.447	0.148	−0.283	0.043
	t4	1.145	0.106	10.811	0.000	0.937	1.353
WHOQOL_12	t1	−0.922	0.097	−9.508	0.000	−1.112	−0.732
	t2	−0.415	0.085	−4.854	1.210×10^{-6}	−0.582	−0.247
	t3	0.044	0.083	0.526	0.599	−0.119	0.206
	t4	1.084	0.103	10.508	0.000	0.882	1.286
WHOQOL_13	t1	−0.668	0.090	−7.427	1.108×10^{-13}	−0.844	−0.491
	t2	−0.231	0.084	−2.761	0.006	−0.395	−0.067
	t3	0.087	0.083	1.053	0.293	−0.075	0.250
	t4	1.124	0.105	10.712	0.000	0.919	1.330
WHOQOL_14	t1	−1.027	0.101	−10.187	0.000	−1.225	−0.830
	t2	−0.322	0.084	−3.810	1.392×10^{-4}	−0.487	−0.156
	t3	0.512	0.087	5.892	3.819×10^{-9}	0.342	0.682
	t4	1.084	0.103	10.508	0.000	0.882	1.286
WHOQOL_23	t1	−1.166	0.107	−10.908	0.000	−1.376	−0.957
	t2	−0.487	0.086	−5.633	1.769×10^{-8}	−0.657	−0.318
	t3	0.164	0.083	1.973	0.048	0.001	0.327
	t4	0.973	0.099	9.853	0.000	0.780	1.167
WHOQOL_24	t1	−1.712	0.146	−11.710	0.000	−1.998	−1.425
	t2	−0.889	0.096	−9.273	0.000	−1.077	−0.701
	t3	−0.439	0.086	−5.114	3.151×10^{-7}	−0.607	−0.271
	t4	0.857	0.095	9.034	0.000	0.671	1.043
WHOQOL_25	t1	−1.307	0.114	−11.421	0.000	−1.531	−1.082
	t2	−0.550	0.088	−6.279	3.415×10^{-10}	−0.721	−0.378
	t3	0.463	0.086	5.374	7.704×10^{-8}	0.294	0.632
	t4	1.257	0.112	11.265	0.000	1.038	1.476
WHOQOL_28	t1	−1.624	0.138	−11.791	0.000	−1.894	−1.354

Thresholds

Indicator	Threshold	Estimate	Std. Error	z-value	p	95% Confidence Interval	
						Lower	Upper
	t2	−1.417	0.121	−11.672	0.000	−1.655	−1.179
	t3	−0.403	0.085	−4.724	2.316×10^{-6}	−0.570	−0.236
	t4	0.109	0.083	1.316	0.188	−0.053	0.272
WHOQOL_29	t1	−2.378	0.260	−9.142	0.000	−2.888	−1.868
	t2	−1.666	0.142	−11.762	0.000	−1.944	−1.389
	t3	−0.512	0.087	−5.892	3.819×10^{-9}	−0.682	−0.342
	t4	5.778×10^{-18}	0.083	6.977×10^{-17}	1.000	−0.162	0.162
WHOQOL_30	t1	−0.614	0.089	−6.919	4.537×10^{-12}	−0.788	−0.440
	t2	−0.142	0.083	−1.710	0.087	−0.305	0.021
	t3	0.379	0.085	4.463	8.086×10^{-6}	0.213	0.546
WHOQOL_31	t1	−1.360	0.118	−11.559	0.000	−1.590	−1.129
	t2	−0.463	0.086	−5.374	7.704×10^{-8}	−0.632	−0.294
	t3	0.055	0.083	0.658	0.511	−0.108	0.217
	t4	0.695	0.091	7.680	1.599×10^{-14}	0.518	0.873
WHOQOL_32	t1	−1.360	0.118	−11.559	0.000	−1.590	−1.129
	t2	−0.627	0.089	−7.047	1.831×10^{-12}	−0.802	−0.453
	t3	0.055	0.083	0.658	0.511	−0.108	0.217
	t4	0.695	0.091	7.680	1.599×10^{-14}	0.518	0.873
WHOQOL_33	t1	−1.333	0.116	−11.493	0.000	−1.560	−1.105
	t2	−0.415	0.085	−4.854	1.210×10^{-6}	−0.582	−0.247
	t3	0.197	0.083	2.367	0.018	0.034	0.361
	t4	0.922	0.097	9.508	0.000	0.732	1.112
WHOQOL_34	t1	−0.391	0.085	−4.593	4.362×10^{-6}	−0.558	−0.224
	t2	0.276	0.084	3.286	0.001	0.111	0.441
	t3	0.973	0.099	9.853	0.000	0.780	1.167
	t4	1.874	0.165	11.370	0.000	1.551	2.198
WHOQOL_35	t1	−1.166	0.107	−10.908	0.000	−1.376	−0.957
	t2	0.033	0.083	0.395	0.693	−0.130	0.195
	t3	0.723	0.091	7.930	2.220×10^{-15}	0.544	0.902
	t4	1.145	0.106	10.811	0.000	0.937	1.353
WHOQOL_36	t1	−0.709	0.091	−7.805	5.995×10^{-15}	−0.887	−0.531
	t2	−0.265	0.084	−3.155	0.002	−0.429	−0.100
	t3	0.575	0.088	6.536	6.335×10^{-11}	0.403	0.748
	t4	1.124	0.105	10.712	0.000	0.919	1.330
WHOQOL_37	t1	−0.322	0.084	−3.810	1.392×10^{-4}	−0.487	−0.156
	t2	0.276	0.084	3.286	0.001	0.111	0.441
	t3	0.973	0.099	9.853	0.000	0.780	1.167
	t4	1.815	0.158	11.522	0.000	1.506	2.123
WHOQOL_38	t1	−1.211	0.109	−11.093	0.000	−1.424	−0.997
	t2	0.011	0.083	0.132	0.895	−0.151	0.173
	t3	0.752	0.092	8.179	2.220×10^{-16}	0.572	0.932
	t4	1.104	0.104	10.611	0.000	0.900	1.308
WHOQOL_39	t1	−0.695	0.091	−7.680	1.599×10^{-14}	−0.873	−0.518
	t2	−0.322	0.084	−3.810	1.392×10^{-4}	−0.487	−0.156
	t3	0.487	0.086	5.633	1.769×10^{-8}	0.318	0.657
	t4	1.046	0.102	10.295	0.000	0.847	1.245

Average variance extracted

Factor	AVE
Factor 1	0.551
Factor 2	0.679
Factor 3	0.740
Factor 4	0.446
Factor 5	0.657
Factor 6	0.828
Factor 7	0.944

Heterotrait-monotrait ratio

Factor 1	Factor 2	Factor 3	Factor 4	Factor 5	Factor 6	Factor 7
1.000						
0.737	1.000					
0.745	0.771	1.000				
0.912	0.839	0.752	1.000			
0.225	0.148	0.294	0.133	1.000		
0.222	0.167	0.248	0.119	0.785	1.000	
0.632	0.517	0.570	0.579	0.229	0.126	1.000

Plots

Misfit plot

	WHOQOL_3	WHOQOL_4	WHOQOL_10	WHOQOL_15	WHOQOL_16	WHOQOL_17	WHOQOL_18	WHOQOL_5	WHOQOL_6	WHOQOL_7	WHOQOL_11	WHOQOL_19	WHOQOL_26	WHOQOL_20	WHOQOL_22	WHOQOL_8	WHOQOL_9	WHOQOL_12	WHOQOL_13	WHOQOL_14	WHOQOL_23
WHOQOL_3	0																				
WHOQOL_4	.17	0																			
WHOQOL_10	.02	.02	0																		
WHOQOL_15	.15	0	.19	0																	
WHOQOL_16	.17	.09	.11	.19	0																
WHOQOL_17	.08	.03	.06	.05	.03	0															
WHOQOL_18	.12	.04	.11	.17	0	.13	0														
WHOQOL_5	.04	.12	.14	.16	.07	.09	.1	0													
WHOQOL_6	.05	.08	.01	.08	.07	.01	.13	.01	0												
WHOQOL_7	.08	.06	.04	.13	.01	.11	.01	.06	.1	0											
WHOQOL_11	.08	.06	.1	.08	.04	.07	.06	.14	.05	.04	0										
WHOQOL_19	.07	.03	.04	.08	.13	.01	.11	.18	.2	.05	.12	0									
WHOQOL_26	.08	.05	.01	.08	.06	.02	.06	.18	.2	.06	.1	.03	0								
WHOQOL_20	.07	.11	.28	0	.16	.01	.01	.15	.02	.02	.17	.07	.04	0							
WHOQOL_22	.12	.03	.04	.07	.01	.01	.03	.17	.13	.05	.1	.01	.03	0	0						
WHOQOL_8	.09	.11	.15	.13	.15	.17	.05	.1	.06	.06	.11	.16	.17	.28	.16	0					
WHOQOL_9	.11	.02	.21	.06	.14	.1	0	.08	.04	.01	.09	.01	.03	.03	.07	.08	0				
WHOQOL_12	.03	.04	.08	.01	.17	.03	.08	.13	.07	.03	.14	.22	.22	.11	.07	.02	.06	0			
WHOQOL_13	.16	.1	.04	.01	.06	.01	.07	.13	.05	.01	.1	.18	.17	.06	.03	.04	.17	.28	0		
WHOQOL_14	.01	.18	.05	.17	.06	.15	.01	.21	.03	.11	.15	.08	.08	.11	.08	.16	.13	.16	.17	0	
WHOQOL_23	.02	.05	.01	.04	.06	.03	.04	.05	.09	.01	.03	.05	.05	.03	.02	.05	.11	.27	.19	.12	0
WHOQOL_24	.02	.14	.01	.01	.03	.07	.01	.16	.12	.03	.18	.01	.01	.21	.2	.09	.06	.08	.05	.09	.07
WHOQOL_25	.08	.15	.05	.14	.04	.1	.06	.11	.04	.03	.05	.05	.06	.02	.02	.1	.13	.13	.16	.06	.01
WHOQOL_28	.14	.02	.07	.01	.05	.01	.11	.13	.02	0	.02	.04	.03	.01	.03	.07	.04	.01	.06	.08	.07
WHOQOL_29	.1	0	.06	.02	.08	.06	.01	.11	.06	.02	.02	.05	.02	.1	.02	.04	.01	.02	.04	.13	.08
WHOQOL_30	.22	.09	.04	.01	.08	0	.02	.03	.1	.03	.02	.03	.03	.01	.04	.05	.06	.02	.08	.07	.16
WHOQOL_31	.2	.07	.02	.01	.11	0	.07	.08	.03	.01	.01	.03	.02	0	.04	.01	.07	.04	.04	.03	.16
WHOQOL_32	.26	.03	.01	.02	.08	.02	.02	0	.08	.13	.05	.02	.05	.01	.04	0	.02	0	.05	.03	.16
WHOQOL_33	.21	.03	.02	.03	.13	.04	.06	.08	.01	.02	.03	.02	.02	.02	.02	.01	.08	.05	.05	.01	.12
WHOQOL_34	.02	.15	.09	.09	.12	.07	.16	.06	.22	0	.06	.02	.02	.05	.07	.2	.07	.06	.11	.07	.07
WHOQOL_35	.1	.09	.13	.17	.25	.02	.12	.09	.11	.13	.03	.05	.05	.04	.04	.14	.1	.12	.01	.17	.25
WHOQOL_36	.13	.01	.21	.02	.18	.05	.06	.12	.05	.05	.02	.14	.11	.02	.05	.07	.19	.05	.13	.13	.1

WHOQOL_37	.02	.14	.06	.1	.1	.08	.17	.06	.2	.01	.08	.02	.02	.08	.06	.22	.06	.05	.13	.07	.08
WHOQOL_38	.11	.1	.15	.17	.23	.01	.1	.05	.13	.08	.05	.04	.03	.05	.04	.18	.09	.05	.05	.16	.24
WHOQOL_39	.18	.06	.2	.03	.17	.01	.04	.11	.07	.01	.02	.12	.08	.03	.08	.03	.13	.01	.1	.1	.09

Model plot

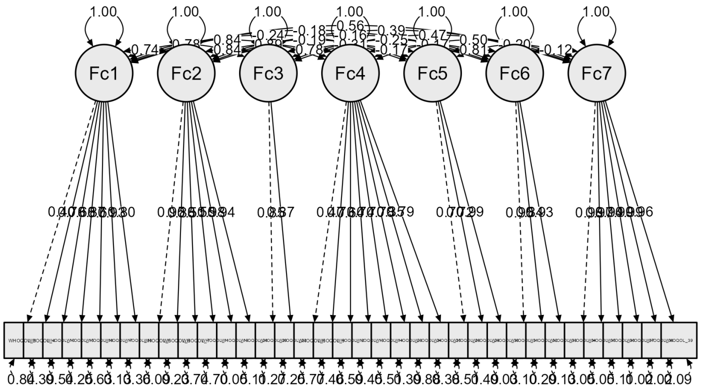

Supplement: Supplementary file 1 [file Data_Sheet_1.pdf]
