# Supplementary material for: Moving toward wellbeing: physical activity and quality of life in individuals with physical disabilities in Saudi Arabia
Source: Front Psychol. 2025 Nov 3;16:1684083. doi: 10.3389/fpsyg.2025.1684083 (PMC12620481; doi:10.3389/fpsyg.2025.1684083)
Supplement: Supplementary file 2 [file Data_Sheet_2.pdf]

Supplementary File 2: Bivariate Correlation Matrix of All Study Variables.

Correlations

		HRA	HHA	SRA	OTA	PH	Psychological_	SRS	Environment	Discrimination	Autonomy	Inclusion
HRA	r	1										
	p											
	N	230										
HHA	r	.795**	1									
	p	.000										
	N	230	230									
SRA	r	.785**	.635**	1								
	p	.000	.000									
	N	230	230	230								
OTA	r	.145*	.502**	.148*	1							
	p	.028	.000	.025								
	N	230	230	230	230							
PH	r	.303**	.388**	.290**	.328**	1						
	p	.000	.000	.000	.000							
	N	230	230	230	230	230						
Psychological	r	.027	.226**	.021	.330**	.698**	1					
	p	.681	.001	.746	.000	.000						
	N	230	230	230	230	230	230					
SRS	r	.008	.191**	-.156*	.216**	.452**	.602**	1				
	p	.909	.004	.018	.001	.000	.000					
	N	230	230	230	230	230	230	230				
Environmental	r	.149*	.242**	.076	.244**	.731**	.685**	.601**	1			

	p	.024	.000	.250	.000	.000	.000	.000				
	N	230	230	230	230	230	230	230	230			
Discrimination	r	.067	.112	.156*	.186**	.232**	.102	.018	.033	1		
	p	.315	.091	.018	.005	.000	.124	.785	.615			
	N	230	230	230	230	230	230	230	230	230		
Autonomy	r	.162*	.264**	.190**	.285**	.214**	.252**	.185**	.146*	-.111	1	
	p	.014	.000	.004	.000	.001	.000	.005	.027	.092		
	N	230	230	230	230	230	230	230	230	230	230	
Inclusion	r	-.352**	-.485**	-.238**	-.322**	-.732**	-.621**	-.615**	-.642**	-.056	-.372**	1
	p	.000	.000	.000	.000	.000	.000	.000	.000	.398	.000	
	N	230	230	230	230	230	230	230	230	230	230	230

**. Correlation is significant at the 0.01 level (2-tailed).

*. Correlation is significant at the 0.05 level (2-tailed).
