# Supplementary material for: Moving toward wellbeing: physical activity and quality of life in individuals with physical disabilities in Saudi Arabia
Source: Front Psychol. 2025 Nov 3;16:1684083. doi: 10.3389/fpsyg.2025.1684083 (PMC12620481; doi:10.3389/fpsyg.2025.1684083)
Supplement: Supplementary file 3 [file Data_Sheet_3.pdf]

Table: Standardized Factor Loadings for WHOQOL-DIS Items across subcomponents

WHOQOL Item	QoL1	QoL2	QoL3	QoL4	QoL5	QoL6	QoL7
WHOQOL_3	0.379						
WHOQOL_4	0.467						
WHOQOL_10	-0.697						
WHOQOL_15	-0.783						
WHOQOL_16	-0.793						
WHOQOL_17	-0.979						
WHOQOL_18	-0.799						
WHOQOL_5		0.939					
WHOQOL_6		0.963					
WHOQOL_7		0.721					
WHOQOL_11		0.676					
WHOQOL_19		0.847					
WHOQOL_26		0.075					
WHOQOL_20			0.800				
WHOQOL_22			1.001				
WHOQOL_8				0.415			
WHOQOL_9				0.648			
WHOQOL_12				0.410			
WHOQOL_13				0.506			
WHOQOL_14				0.652			
WHOQOL_23				0.852			
WHOQOL_24				0.800			
WHOQOL_25				0.946			
WHOQOL_28					0.730		
WHOQOL_29					0.845		
WHOQOL_30					1.010		
WHOQOL_31						0.738	
WHOQOL_32						-0.870	
WHOQOL_33						0.889	
WHOQOL_34							0.756
WHOQOL_35							0.811
WHOQOL_36							-0.953
WHOQOL_37							0.761
WHOQOL_38							0.690
WHOQOL_39							0.713
