# Supplementary material for: Moving toward wellbeing: physical activity and quality of life in individuals with physical disabilities in Saudi Arabia
Source: Front Psychol. 2025 Nov 3;16:1684083. doi: 10.3389/fpsyg.2025.1684083 (PMC12620481; doi:10.3389/fpsyg.2025.1684083)
Supplement: Supplementary file 4 [file Data_Sheet_4.pdf]

Supplementary Table S5. Structural model: Standardized associations with the latent overall QoL factor, with 95% confidence intervals.

Predictor	Model 1: Total PASIPD-AR			Model 2: PASIPD-AR Factors		
	β	95% CI	p-value	β	95% CI	p-value
**Self-Perception & Demographics**						
SP-health	0.454	[0.312, 0.596]	<0.001	0.439	[0.298, 0.580]	<0.001
SP-fitness	0.326	[0.185, 0.467]	<0.001	0.387	[0.249, 0.525]	<0.001
Sex (Female)	0.151	[0.025, 0.277]	0.019	0.125	[0.002, 0.248]	0.048
BMI	-0.205	[-0.346, -0.064]	0.005	-0.155	[-0.311, 0.001]	0.053
Age	-0.134	[-0.271, 0.003]	0.055	-0.105	[-0.250, 0.040]	0.101
**Physical Activity**						
Total PASIPD-AR Score	-0.005	[-0.137, 0.127]	0.922	—	—	—
Home Repair Activities	—	—	—	0.074	[-0.053, 0.201]	0.246
Household Activities	—	—	—	-0.114	[-0.242, 0.014]	0.081
Sports & Recreational Activities	—	—	—	0.121	[0.019, 0.223]	0.020
Occupational & Transportation Activities	—	—	—	-0.017	[-0.151, 0.117]	0.814
**Other demographics**	ns	—	>0.05	ns	—	>0.05
