# Supplementary material for: Moving toward wellbeing: physical activity and quality of life in individuals with physical disabilities in Saudi Arabia
Source: Front Psychol. 2025 Nov 3;16:1684083. doi: 10.3389/fpsyg.2025.1684083 (PMC12620481; doi:10.3389/fpsyg.2025.1684083)
Supplement: Supplementary file 5 [file Data_Sheet_5.pdf]

Supplementary Table S7. Associations of demographics, SP-health, SP-fitness, and total PASIPD-AR score with WHOQOL-DIS-AR components, including 95% confidence intervals.

Predictor	Physical Health	Psychological	Social Relationships	Environmental	Discrimination	Autonomy	Inclusion/Participation
**Age**	↑0.208*** (S) [0.102, 0.314]	↓–0.266*** (S) [–0.379, –0.153]	ns	ns	ns	↓–0.130† (S) [–0.275, 0.015]	↑0.182** (S) [0.056, 0.308]
**Sex (Female)**	ns	ns	ns	ns	ns	↑0.107* (S) [0.012, 0.202]	ns
**BMI**	ns	↓–0.122* (S) [–0.233, –0.011]	ns	↓–0.094† (N) [–0.199, 0.011]	ns	ns	ns
**Education level**	↓–0.099† (N) [–0.203, 0.006]	ns	↓–0.328*** (M) [–0.442, –0.214]	ns	↑0.135* (S) [0.027, 0.243]	↑0.140* (S) [0.032, 0.248]	↑0.236*** (S) [0.122, 0.350]
**Occupation**	↓–0.138* (S) [–0.251, –0.025]	↑0.127* (S) [0.022, 0.232]	↑0.226* (S) [0.108, 0.344]	ns	↓–0.235*** (S) [–0.339, –0.131]	↑0.226** (S) [0.093, 0.359]	↓–0.422*** (M) [–0.543, –0.301]
**Type of disability**	↑0.124** (S) [0.044, 0.204]	↓–0.146*** (S) [–0.227, –0.065]	↑0.175** (S) [0.068, 0.282]	ns	ns	↓–0.140* (S) [–0.246, –0.034]	ns
**Social relationships**	↓–0.105* (S) [–0.187, –0.023]	↑0.193*** (S) [0.091, 0.295]	ns	ns	↓–0.162† (S) [–0.334, 0.010]	↑0.265*** (M) [0.145, 0.385]	↓–0.091† (N) [–0.199, 0.017]
**Income**	ns	↑0.091† (N) [–0.003, 0.185]	ns	ns	↓–0.218*** (S) [–0.330, –0.106]	ns	↑0.070† (N) [–0.012, 0.152]
**Disability aid**	ns	↑0.103* (S) [0.017, 0.189]	ns	ns	↓–0.129† (S) [–0.264, 0.006]	ns	↓–0.155** (S) [–0.257, –0.053]
**SP-health**	↓–0.310*** (S) [–0.418, –0.202]	↑0.171*** (S) [0.083, 0.259]	↑0.311** (M) [0.129, 0.493]	↑0.374*** (M) [0.270, 0.478]	ns	↑0.172** (S) [0.068, 0.276]	↓–0.224*** (S) [–0.336, –0.112]
**SP-fitness**	↓–0.288*** (S) [–0.382, –0.194]	↑0.515*** (L) [0.405, 0.625]	↑0.456*** (M) [0.352, 0.560]	↑0.341*** (M) [0.239, 0.443]	↑0.147* (S) [0.031, 0.263]	↑0.156** (S) [0.052, 0.260]	↓–0.365*** (M) [–0.481, –0.249]
**Total PASIPD-AR score**	ns	↓–0.109* (S) [–0.197, –0.021]	ns	↓–0.106† (S) [–0.214, 0.002]	↑0.352*** (M) [0.228, 0.476]	ns	ns
