# Supplementary material for: Moving toward wellbeing: physical activity and quality of life in individuals with physical disabilities in Saudi Arabia
Source: Front Psychol. 2025 Nov 3;16:1684083. doi: 10.3389/fpsyg.2025.1684083 (PMC12620481; doi:10.3389/fpsyg.2025.1684083)
Supplement: Supplementary file 6 [file Data_Sheet_6.pdf]

Table S9. Associations of demographics, SP-health and SP-fitness, and PASIPD-AR factors with specific WHOQOL-DIS-AR domains and facets among IWPDs, with standardized regression coefficients (β), 95% confidence intervals (CIs), and significance levels.

Predictor	Physical Health β (95% CI)	Psychological β (95% CI)	Social Relationships β (95% CI)	Environmental β (95% CI)	Discrimination β (95% CI)	Autonomy β (95% CI)	Inclusion/Participation β (95% CI)
Age	0.193*** (0.115, 0.271)	−0.261*** (−0.340, −0.182)	ns	ns	ns	−0.128† (−0.259, 0.003)	0.173** (0.065, 0.281)
Sex (Female)	ns	−0.067† (−0.145, 0.012)	ns	ns	ns	0.114* (0.012, 0.216)	ns
BMI	ns	−0.111* (−0.196, −0.026)	ns	−0.126* (−0.223, −0.029)	ns	ns	ns
Education level	ns	ns	−0.336*** (−0.425, −0.247)	ns	0.141* (0.031, 0.251)	0.143* (0.034, 0.252)	0.263*** (0.170, 0.356)
Occupation	−0.124* (−0.227, −0.021)	0.149* (0.041, 0.257)	ns	ns	−0.194** (−0.320, −0.068)	ns	−0.390*** (−0.504, −0.276)
Type of disability	0.118** (0.043, 0.193)	−0.165*** (−0.243, −0.087)	0.179** (0.070, 0.288)	ns	ns	−0.129* (−0.234, −0.024)	ns
Social relationships	ns	0.174** (0.061, 0.287)	ns	ns	−0.152* (−0.272, −0.032)	0.261*** (0.166, 0.356)	ns
Income	ns	0.111* (0.020, 0.202)	ns	ns	−0.199** (−0.318, −0.080)	ns	0.077† (−0.009, 0.163)
Disability aid	ns	0.121* (0.024, 0.218)	ns	ns	−0.144* (−0.260, −0.028)	0.154* (0.046, 0.262)	−0.214*** (−0.318, −0.110)
SP-health	−0.325*** (−0.415, −0.235)	0.198*** (0.100, 0.296)	0.295*** (0.193, 0.397)	0.363*** (0.262, 0.464)	ns	0.188** (0.071, 0.305)	−0.233*** (−0.340, −0.126)
SP-fitness	−0.257*** (−0.337, −0.177)	0.479*** (0.391, 0.567)	0.340*** (0.237, 0.443)	0.308*** (0.200, 0.416)	0.193* (0.040, 0.346)	ns	−0.271*** (−0.377, −0.165)

Home Repair Activities	0.442*** (0.337, 0.547)	−0.377*** (−0.484, −0.270)	−0.446*** (−0.552, −0.340)	−0.259** (−0.421, −0.097)	ns	−0.294*** (−0.410, −0.178)	0.353*** (0.247, 0.459)
Household Activities	−0.336*** (−0.448, −0.224)	0.251*** (0.143, 0.359)	0.542*** (0.440, 0.644)	0.149* (0.028, 0.270)	ns	0.531*** (0.420, 0.642)	−0.399*** (−0.505, −0.293)
Sports & Recreational Activities	−0.087† (−0.186, 0.012)	ns	ns	ns	0.288*** (0.151, 0.425)	ns	ns
Occupational & Transportation Activities	−0.105† (−0.206, −0.004)	−0.080† (−0.173, 0.013)	ns	0.081† (−0.010, 0.172)	ns	0.025† (−0.061, 0.111)	−0.060† (−0.152, 0.032)
